# Discovery of Triazone Derivatives Containing Acylhydrazone and Phenoxypyridine Motifs as Novel Insecticidal and Antiphytopathogenic Fungus Agents

**DOI:** 10.3390/ijms27010260

**Published:** 2025-12-26

**Authors:** Peipei Cui, Yan Yang

**Affiliations:** 1 College of Architecture and Arts, Taiyuan University of Technology, Jinzhong 030060, China; cuipeipei@tyut.edu.cn; 2 College of Chemistry and Chemical Engineering, Taiyuan University of Technology, Taiyuan 030024, China

**Keywords:** triazone, hydrazone, phenoxypyridine, biological activities

## Abstract

A series of novel triazone derivatives containing acylhydrazone and phenoxypyridine motifs were designed, synthesized, and evaluated for their biological activities. The bioassay results indicated that most of the target compounds exhibited excellent insecticidal activities against bean aphids. In particular, compounds **3i** and **3e** showed excellent aphicidal activities comparable to pymetrozine, thus emerging as novel insecticidal lead compounds. Additionally, compounds **3c** (60%), **3e** (60%), and **3f** (60%) exhibited good larvicidal activities against C. pipiens pallens at 0.5 mg/kg. Further fungicidal activity tests revealed that most derivatives exhibited broad-spectrum fungicidal activities. A total of twelve compounds exhibited better fungicidal activities against cercospora arachidicola hori than carbendazim, and eight compounds exhibited better fungicidal activities against fusarium moniliforme than carbendazim. This work suggests that compound **3e** could serve as an insecticidal lead compound for further structural optimization.

## 1. Introduction

Agricultural disasters induced by phytophagous aphids can cause great losses in agricultural production [1]. There are about 100 species of aphids all over the world, which are very difficult to control due to their extremely short life cycle and high reproductive rate [2]. At present, chemical aphicides (carbamates, organophosphorus compounds, and neonicotinoids) remain the primary measure for effectively preventing agricultural disasters. However, widespread and prolonged use of chemical aphicides may lead to resistance and thus become ineffective [3]. Therefore, there is an urgent need to develop novel and efficient green insecticides to control aphids.

**Figure 1:**
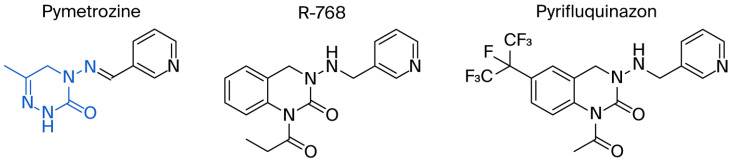
Pymetrozine, R-768, and pyrifluquinazon.

Pymetrozine (Figure 1), a triazone insecticide discovered by Syngenta in 1988, exhibited good activities against aphids, white flies, and planthoppers [4]. Since then, such insecticides have attracted great research attention for decades [5,6,7,8,9,10,11]. Our previous studies have also indicated that triazone derivatives containing phenoxypyridine, urea, sulfonamide or sulfonimide, acylhydrazone display significant insecticidal properties [12,13,14,15]. However, only two other commercial insecticides (R-768 and pyrifluquinazon, Figure 1) have been developed so far, the activities of which are significantly lower than that of pymetrozine [16].

Nesterov et al. found that pymetrozine acted on Transient receptor potential channels (TRP channels) [17], which are a super-family of ion channels sensitive to diverse chemical and physical stimuli and which play diverse roles in biology [18]. TRP channels are classified into six subfamilies: TRP canonical, TRP vanilloid, TRP melastatin, TRP ankyrin, TRP polycystin, and TRP mucoliptin [19]. TRPC5 belongs to the canonical subgroup of the TRP super-family. It is a nonselective cation channel that can form many homo- and heterotetrameric channels, which are predominantly expressed in the brain and kidney and can be activated by chemical agents such as compound **1** [20]. Compound **1** is a highly selective, potent TRPC5 antagonist, which may become an important pharmacological tool for research in the pathogenesis of kidney disease [21].

Acylhydrazone plays an important role in pesticide and drug molecular design because of its unique physicochemical properties and outstanding biological activity.

**Figure 2:**
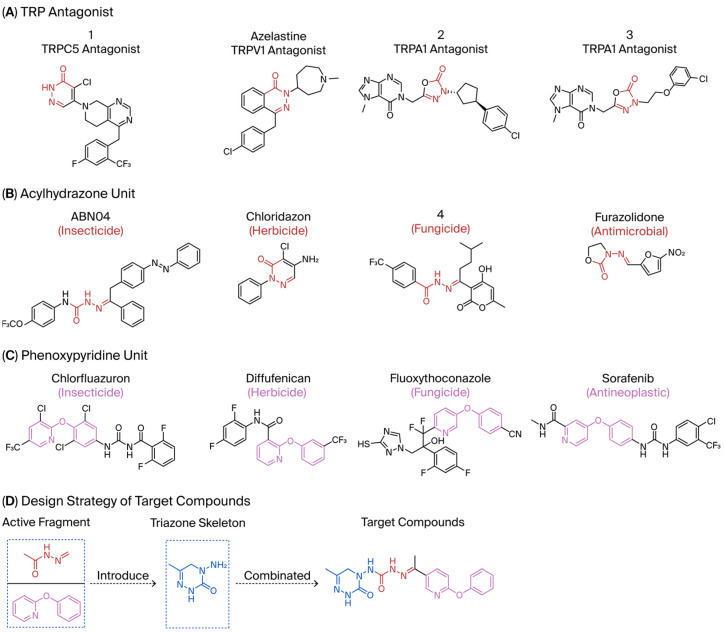
(**A**) TRP antagonist. (**B**) Acylhydrazone unit. (**C**) Phenoxypyridine unit. (**D**) Design strategy of target compounds.

Compound **1**, azelastine [22], and compounds **2** and **3** [23] (Figure 2A) containing acylhydrazone moieties are used as TRP antagonists. ABM04, chloridazon, compound **4**, and furazolidone (Figure 2B), which contain acylhydrazone moieties, are used as insecticide [24], herbicide [25], fungicide [26], and antituberculosis [27] agents, respectively.

Phenoxypyridine (Figure 2C), the bioisostere of diaryl ethers, has been widely introduced into bioactive molecules and has demonstrated significant biological activities. Chlorfluazuron, diflufenican, fluoxytioconazole, and sorafenib containing phenoxypyridine moieties exhibited insecticidal [28], herbicidal [29], and antifungal [30] activities, respectively.

Based on the above considerations, through the principle of activity splicing, acylhydrazone and phenoxypyridine active moieties were introduced into a triazone core skeleton to synthesize a series of novel triazone derivatives. Their insecticidal and fungicidal activities were evaluated.

## 2. Results and Discussion

### 2.1. General Synthesis

**Figure 3:**
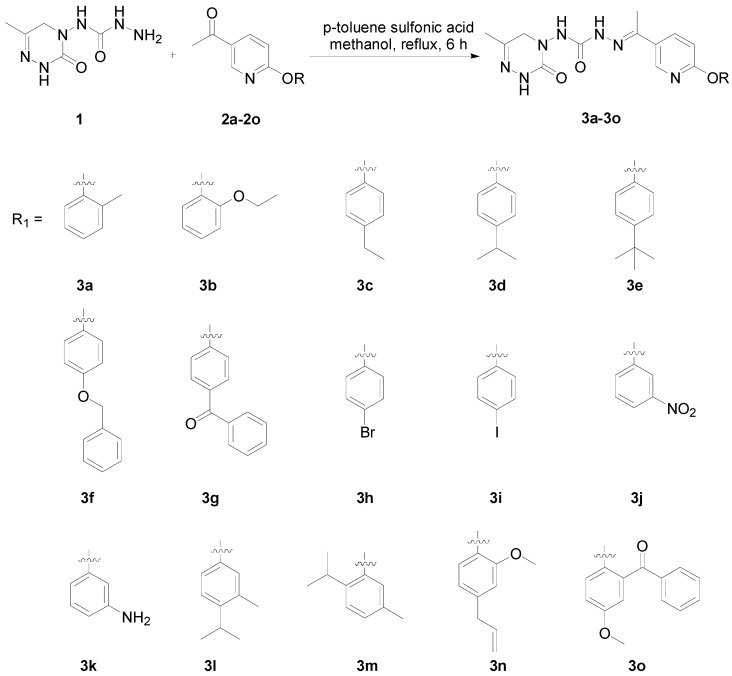
Synthesis of compounds **3a**–**3o**.

The synthetic routes of target compounds **3a**–**3o** are shown in Figure 3. Intermediates **1** and **2** were synthesized according to the literature [12,13]. The condensation of triazinone **A** and compound **B** in 1,2-dichloroethane afforded ester **C**, which reacted with hydrazine hydrate in methanol to yield compound **1**. Compounds **3a**–**3o** were synthesized by the condensation of compound **1** and phenoxypyridines **2a**–**2o** under the catalysis of *p*-toluenesulfonic acid in 22–88% yields. Most target products had high yields; only the yields of compounds **3f**, **3g**, **3m**, and **3o** were lower than 50%. ^1^H NMR, ^13^C NMR, and HRMS data of compounds **3a**–**3o** are available in the Supplementary Materials.

### 2.2. Biological Evaluation

#### 2.2.1. Foliar Contact Activity Against Bean Aphid (*A. craccivora*)

Table 1 shows that most target compounds exhibited good insecticidal activities. Among them, compounds **3b** (75%), **3c** (70%), **3d** (80%), **3e** (90%), **3h** (70%), **3i** (80%) and **3l** (75%) still exhibited excellent biological activities at 10 mg/kg. In particular, compounds **3i** (80%) and **3e** (90%) exhibited the same level of activities as pymetrozine (90%). The activity was moderate compared to literature data [12,13,14,15]. The size of the substituents on the phenoxy group significantly influenced aphicidal activities (**3c**–**3e**). For example, an analysis of the structure–activity relationship indicated aphicidal activities in the following order: **3e** *(p*-tert-butyl, 90%) > **3d** (*p*-isopropyl, 80%) > **3c** (*p*-ethyl, 70%). The position of substituents on the phenoxy ring had a significant influence on aphicidal activities; for example, compound **3l** (4-isopropyl, 75%) exhibited higher activity than **3m** (2-isopropyl, 30%) at 10 mg/kg. At 5mg/kg, the activity of *p*-tert-butyl substituted compound against Bean Aphids is the highest, followed by *p*-iodine substituted compound and then *p*-isopropyl substituted compound. The introduction of electron-withdrawing groups and electron-donating groups will not lead to a significant increase in insecticidal activity.

**Figure 4:**
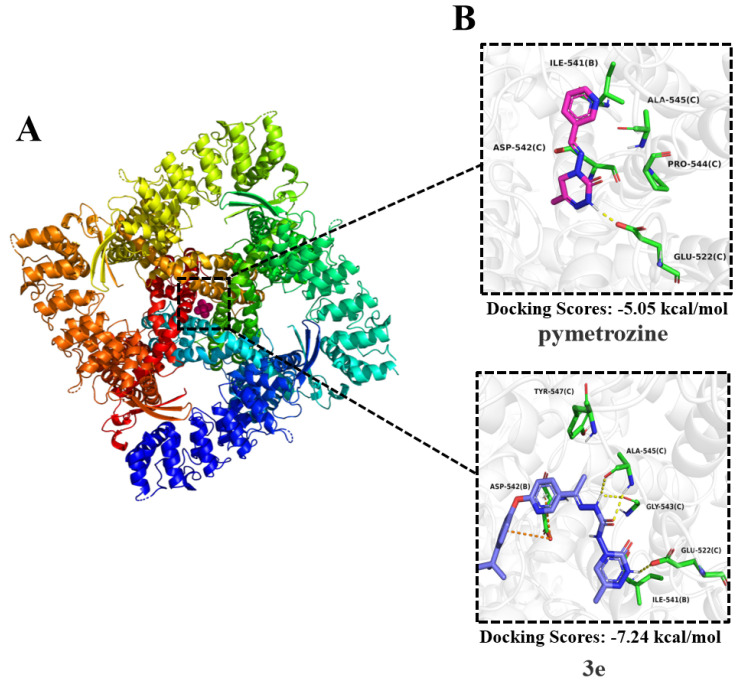
(**A**) The rabbit TRPV protein. (**B**) Molecular docking diagrams of compound **3e** and pymetrozine with the rabbit TRPV protein.

The rabbit TRPV protein was selected as a homology for molecular docking studies. The target compound was **3e**, with pymetrozine serving as the positive control. The protein forms a homotetramer consisting of four subunits (A, B, C, and D). The binding site was defined as the central cavity near the crystallographic ligand Ruthenium Red, a region previously identified in published structures. Within this site, three key residues, Ser542, Thr539 and Asn572, were selected to delineate the binding pocket (Figure 4A).

The results (Figure 4B) show that pymetrozine, due to its smaller size, binds closer to the B and C subunits. Its triazine ring forms a hydrogen bond with Glu522 (subunit C), while also engaging in hydrophobic interactions with Ile541 (B), Asp542 (C), Pro544 (C), and Ala545 (C). In contrast, compound **3e**, which incorporates a diaryl group that increases its molecular volume, showed superior complementarity to the binding pocket. The introduced diaryl moiety engages in a π-anion interaction with Asp542 (subunit B). Furthermore, the triazine ring maintains the hydrogen bond with Glu522 (C), while the acyl hydrazone group forms additional hydrogen bonds with Gly543 (C) and Ala545 (C). Compared to pymetrozine, **3e** benefits from a greater number of hydrogen bonds and the new π-anion interaction, collectively contributing to stronger target binding. This is corroborated by the molecular docking scores: −7.24 kcal/mol for **3e** versus −5.05 kcal/mol for pymetrozine. The enhanced binding affinity provides a structural rationale for the superior insecticidal activity of **3e** (Figure 4B).

Based on the molecular docking, it is speculated that the molecule of pymetrozine was relatively small compared to the TRPV5 channel protein binding pocket. This resulted in pymetrozine being unable to effectively bind to the receptor, leading to a low docking score for pymetrozine. Therefore, increasing the molecular volume of molecules and introducing new effective functional groups is key to enhancing the insecticidal activity. By introducing a diaryl fragment, the volume of compound **3e** significantly increased. Therefore, compound **3e** exhibited insecticidal activity against *A. craccivora* similar to that of pymetrozine. It also exhibited higher insecticidal activity against *C. pipiens pallens* than pymetrozine. Subsequent design of compounds can consider introducing large functional groups.

#### 2.2.2. Larvicidal Activities

The larvicidal activities of compounds **3a**–**3o** and pymetrozine against *C. pipiens pallens, H. armigera, M. separata*, and *O. nubilalis* are listed in Table 2 and Table 3. The larvicidal activities of compounds **3a**, **3c**, **3e**, and **3f** against *C. pipiens pallens* were higher than that of pymetrozine. Compounds **3c** (*p*-ethyl), **3e** (*p-tert*-butyl), and **3f** (*p*-benzyloxy) showed higher larvicidal activities (60% at 0.5 mg/kg) than that of pymetrozine. Most alkyl substituents exhibited better activities against *C. pipiens pallens* than other electron withdrawing and electron donating substituents. The larvicidal activity of compound **3a** (*o*-methyl) against *H. armigera*, *O. nubilalis*, and *M. separata* was the highest among the 15 compounds.

#### 2.2.3. Fungicidal Activities

Compounds **3a**–**3o** were also evaluated for their fungicidal activities at 50 mg/kg, with two commercial fungicides, carbendazim and chlorothalonil, used as controls (Table 4). These derivatives showed broad-spectrum fungicidal activities against 14 kinds of plant fungi, with particularly excellent fungicidal activities against *Physalospora piricola* and *Rhizoctonia cerealis*. Additionally, all compounds showed better fungicidal activities against *Physalospora piricola* than carbendazim. Twelve compounds showed better fungicidal activities against *Cercospora arachidicola hori* than carbendazim, while eight compounds showed better fungicidal activities against *Fusarium moniliforme* than carbendazim. Compound **3b** showed better fungicidal activity against *Fusarium graminearum* than chlorothalonil and exhibited an inhibition rate greater than 50% against eight kinds of fungi. Compounds **3d**, **3g**, **3m**, and **3n** exhibited an inhibition rate greater than 50% against six kinds of fungi.

#### 2.2.4. Toxicity

The acute, mutagenic and carcinogenic toxicities of compounds **3a**–**3o** are listed in Table 5, which were predicted by the Pro Tox-3.0 program. The predicted acute toxicities of compounds **3a**–**3o** are relatively low. The mutagenic and carcinogenic toxicities of compound **3e** are negative. Based on the activity and low toxicity of compound **3e**, it can be used as a lead compound for further structural optimization.

## 3. Materials and Methods

**Instruments.** ^1^H and ^13^C NMR spectra were obtained using a Bruker AV400 spectrometer (Bruker Corporation, Billerica, MA, USA) in DMSO-*d*_6_, while HRMS data were obtained using an FTICR-MS instrument (Ionspec 7.0 T, Ionspec Corporation, San Diego, CA, USA). The melting points were tested on an X-4 binocular microscope (Beijing Tech Instruments Co., Beijing, China) melting point apparatus.

**General synthesis.** Pymetrozine was purchased from Chemieliva Pharmaceutical Co. (Chongqing, China) and used as the control in the insecticidal activity tests. Chlorothalonil and carbendazim, which were used in the bactericidal activity tests, were purchased from Bailing Agrochemical Co., Ltd. (Taizhou, China). All reagents were purchased and used directly, and all solvents were dried using standard techniques reported in the literature. 

**General Procedure for the Preparation of Compounds 3a–3o.** To a solution of compound 1 (4.00 mmol) and compounds **2a**–**2o** (4.00 mmol) in methanol (80 mL) was added *p*-toluene sulfonic acid (0.80 mmol), and then the mixture was refluxed for 6 h. The solution was cooled and then concentrated under reduced pressure. The crude product was purified by flash chromatography on silica gel using petroleum ether (60–90 °C) and ethyl acetate (*v*/*v* = 5:1) as eluent to give compound **3a**–**3o** as white solids.

**Biological Evaluation.** The larvicidal activities of **3a**–**3o** were tested using previously reported methods [31,32,33], while fungicidal activities were tested using a previously reported procedure [34] with pymetrozine as control. The testing process is recorded in detail in the Supplementary Materials.

**Molecular docking.** Molecular docking was performed using the Molecular Operating Environment (MOE) software (version 2024.06). The molecular docking studies of target compound **3e** were conducted with pymetrozine serving as positive control. The rabbit TRPV protein was selected as a homolog for molecular docking studies. The protein crystal structure for molecular docking was selected from the Protein Data Bank (http://www.rcsb.org/) with the PDB ID 8FFN. This structure corresponds to the rabbit TRPV5 channel protein. According to the literature, the crystal contains the ligand ruthenium red, and the binding region of ruthenium red was defined as the protein binding pocket. Based on prior reports, residues Thr539 and Asp542 were identified as key amino acids for ligand binding, and the region surrounding these two residues was selected as the docking site.

Prior to docking, the protein structure was checked and optimized using the Structure Preparation module. The target compounds were energy-minimized using the Amber10:ETH force field. The highest-scoring binding conformation of each compound was used as the initial input. Docking was carried out with the Triangle Matcher placement method at the defined binding site. Semi-flexible docking was conducted using the GBVI/WSA dG scoring function, and 30 docking poses were generated and ranked by score. Finally, cluster analysis was performed using an RMSD threshold of 2.0 Å to select the best-scored pose for each compound. Molecular docking figures were generated using PyMOL v1.8.

**Prediction of toxicity** Pro Tox-3.0 is a program used to predict acute toxicity, mutagenic toxicity and carcinogenic toxicity of organic compounds, the URL of which is https://tox.charite.de. The prediction results of acute toxicity correspond to rat, oral and range of LD_50_.

## 4. Conclusions

In summary, a series of novel triazone derivatives containing acylhydrazone and phenoxypyridine groups were synthesized and evaluated for their insecticidal and fungicidal activities. Compounds **3i** and **3e** exhibited aphicidal activities comparable to pymetrozine and could be used as lead compounds for further research. According to the results of molecular docking, compound **3e** can form more hydrogen bond and π-anion ion interactions with rabbit TRPV protein compared to pymetrozine, which can be confirmed by the molecular docking scores of compound **3e** being −7.24 kcal/mol and pymetrozine being −5.05 kcal/mol. The enhanced binding affinity provides a structural rationale for the superior insecticidal activity of **3e**.

Compounds **3c**, **3e**, and **3f** exhibited 60% insecticidal activities against *C. pipiens pallens* at 0.5 mg/Kg. A total of 12 compounds exhibited better fungicidal activities against *Cercospora arachidicola hori* than carbendazim, and 8 compounds exhibited better fungicidal activities against *Fusarium moniliforme* than carbendazim. Further studies on these triazone compounds are in progress in our laboratory.

## Figures and Tables

**Table 1:** Foliar contact activities against *A. craccivora* of compounds **3a**–**3o** and pymetrozine ^a^.

Compound	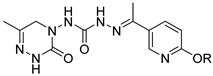 R	Mortality (%) at Concentration (mg/kg)			
		600	100	10	5
**3a**	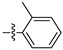	100 ± 0	100 ± 0	40 ± 0	-
**3b**	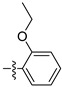	100 ± 0	90 ± 0	75 ± 2	10 ± 0
**3c**	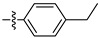	100 ± 0	95 ± 1	70± 0	10 ± 0
**3d**	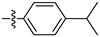	100 ± 0	100 ± 0	80 ± 0	15 ± 1
**3e**	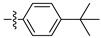	100 ± 0	100 ± 0	90 ± 0	35 ± 2
**3f**	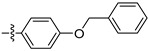	100 ± 0	90 ± 0	45 ± 2	-
**3g**	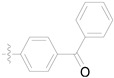	100 ± 0	85 ± 1	25 ± 2	-
**3h**	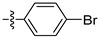	100 ± 0	100 ± 0	70 ± 0	10 ± 0
**3i**	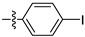	100 ± 0	95 ± 2	80 ± 0	20 ± 0
**3j**	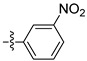	100 ± 0	80 ± 0	30 ± 0	-
**3k**	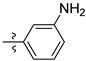	100 ± 0	90 ± 0	20 ± 0	-
**3l**	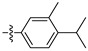	100 ± 0	95 ± 2	75 ± 1	10 ± 0
**3m**	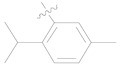	100 ± 0	85 ± 2	30 ± 0	-
**3n**	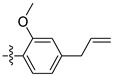	100 ± 0	80 ± 0	20 ± 0	-
**3o**	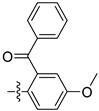	100 ± 0	90 ± 0	35 ± 2	-
pymetrozine	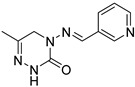	100 ± 0	100 ± 0	90 ± 0	30 ± 0

^a^ Average of three replicates; All results are expressed as mean ± SD; Statistical analysis was conducted using SPSS 27.0 software. Statistical significance was defined as *p* < 0.05. - Not tested.

**Table 2:** Larvicidal activities of compounds **3a**–**3o** and pymetrozine against *C. pipiens pallens* ^a^.

Compound	Correcting Mortality (%) at Concentration (mg/kg)				
	10	5	2	1	0.5
**3a**	100 ± 0	100 ± 0	100 ± 0	100 ± 0	40 ± 0
**3** **b**	100 ± 0	40 ± 0	-	-	-
**3** **c**	100 ± 0	100 ± 0	100 ± 0	100 ± 0	60 ± 0
**3** **d**	100 ± 0	20 ± 0	-	-	-
**3** **e**	100 ± 0	100 ± 0	100 ± 0	100 ± 0	60 ± 0
**3** **f**	100 ± 0	100 ± 0	100 ± 0	100 ± 0	60 ± 0
**3** **g**	40 ± 0	-	-	-	-
**3** **h**	20 ± 0	-	-	-	-
**3** **i**	100 ± 0	30 ± 0	-	-	-
**3** **j**	100 ± 0	30 ± 0	-	-	-
**3** **k**	100 ± 0	100 ± 0	60 ± 0	-	-
**3** **l**	30 ± 0	-	-	-	-
**3** **m**	100 ± 0	100 ± 0	60 ± 0	-	-
**3** **n**	100 ± 0	40 ± 0	-	-	-
**3** **o**	70 ± 0	-	-	-	-
pymetrozine	100 ± 0	40 ± 0	-	-	-

^a^ Average of three replicates; All results are expressed as mean ± SD; Statistical analysis was conducted using SPSS 27.0 software. Statistical significance was defined as *p* < 0.05. - Not tested.

**Table 3:** Larvicidal activities of compounds **3a**–**3o** and pymetrozine against *H. armigera*, *O. nubilalis*, and *M. separata* ^a^.

Compound	Correcting Mortality (%) at Concentration 600 mg/kg		
	*H. armigera*	*O. ubilalis*	*M. eparata*
**3a**	45 ± 2	40 ± 0	65 ± 1
**3b**	35 ± 1	30 ± 0	45 ± 2
**3c**	0	0	5 ± 0
**3d**	20 ± 0	10 ± 0	20 ± 0
**3e**	15 ± 1	10 ± 0	20 ± 0
**3f**	0	0	5 ± 0
**3g**	10 ± 0	5 ± 1	20 ± 0
**3h**	15 ± 1	10 ± 0	20 ± 0
**3i**	40 ± 0	35 ± 2	65 ± 2
**3j**	10 ± 0	5 ± 0	10 ± 0
**3k**	40 ± 0	35 ± 2	50 ± 0
**3l**	20 ± 0	15 ± 1	20 ± 0
**3m**	30 ± 0	25 ± 2	45 ± 2
**3n**	20 ± 0	15 ± 1	25 ± 0
**3o**	40 ± 0	35 ± 2	65 ± 2
pymetrozine	20 ± 0	40 ± 0	50 ± 0

^a^ Average of three replicates; All results are expressed as mean ± SD; Statistical analysis was conducted using SPSS 27.0 software. Statistical significance was defined as *p* < 0.05.

**Table 4:** In vitro fungicidal activities of compounds **3a**–**3o**, carbendazim, and chlorothalonil against 14 kinds of fungi ^a^.

Compound	Fungicidal Activity (%) ^a^ at 50 mg/kg													
	AS ^b^	FG	PI	PC	SS	BC	RS	FC	CH	PP	RC	BM	WA	FM
**3a**	18.8 ± 0.6	38.5 ± 1.4	4.8 ± 2.2	24.1 ± 1.9	12.1 ± 0.7	18.4 ± 0.5	10.5 ± 1.8	60.0 ± 2.3	60.0 ± 0.7	80.3 ± 0.9	74.1 ± 2.0	50.0 ± 1.1	39.3 ± 0.9	46.7 ± 0.6
**3b**	18.8 ± 1.3	57.7 ± 0.5	23.8 ± 1.4	31.0 ± 0.7	20.7 ± 1.3	10.5 ± 0.9	68.4 ± 1.5	52.5 ± 1.8	56.0 ± 0.9	82.0 ± 1.3	80.2 ± 2.6	60.0 ± 3.4	50.0 ± 1.6	63.3 ± 1.7
**3c**	12.5 ± 2.5	38.5 ± 1.8	9.5 ± 1.9	31.0 ± 1.5	24.1 ± 1.7	13.2 ± 0.5	14.0 ± 0.7	37.5 ± 1.4	52.0 ± 2.1	72.1 ± 1.6	72.8 ± 2.4	47.5 ± 0.6	50.0 ± 0.9	60.0 ± 2.2
**3d**	12.5 ± 0.8	46.2 ± 1.8	4.8 ± 1.2	27.6 ± 0.9	20.7 ± 0.7	10.5 ± 0.4	22.8 ± 0.5	67.5 ± 0.7	56.0 ± 0.9	73.8 ± 1.6	79.0 ± 2.1	57.5 ± 2.3	53.6 ± 0.9	50.0 ± 0.7
**3e**	25.0 ± 1.2	11.5 ± 0.9	9.5 ± 0.7	17.2 ± 0.8	24.1 ± 1.3	26.3 ± 1.3	33.3 ± 1.8	47.5 ± 2.2	52.0 ± 2.1	82.0 ± 0.6	80.2 ± 1.2	57.5 ± 2.1	42.9 ± 1.5	60.0 ± 2.4
**3f**	6.3 ± 0.9	19.2 ± 0.7	4.8 ± 2.1	24.1 ± 0.7	12.1 ± 0.8	10.5 ± 0.5	40.4 ± 0.8	40.0 ± 2.2	44.0 ± 2.6	73.8 ± 1.7	79.0 ± 1.5	60.0 ± 2.7	35.7 ± 1.1	33.3 ± 0.3
**3g**	6.3 ± 1.1	11.5 ± 0.5	14.3 ± 0.7	10.3 ± 0.9	12.1 ± 0.3	7.9 ± 0.6	10.5 ± 2.6	57.5 ± 1.9	56.0 ± 0.6	83.6 ± 0.9	82.7 ± 1.3	50.0 ± 1.7	57.1 ± 2.1	66.7 ± 2.5
**3h**	12.5 ± 1.4	30.8 ± 0.5	9.5 ± 0.5	31.0 ± 1.7	20.7 ± 0.7	18.4 ± 2.4	10.5 ± 1.6	35.0 ± 1.2	60.0 ± 1.5	80.3 ± 2.7	82.7 ± 1.9	55.0 ± 2.5	46.4 ± 1.5	50.0 ± 0.8
**3i**	12.5 ± 0.8	46.2 ± 2.3	9.5 ± 0.9	34.5 ± 0.7	20.7 ± 1.3	10.5 ± 0.5	31.6 ± 0.8	45.0 ± 1.9	56.0 ± 2.5	85.2 ± 1.7	80.2 ± 2.1	62.5 ± 1.2	50.0 ± 0.7	46.7 ± 0.9
**3j**	12.5 ± 0.8	23.1 ± 0.7	4.8 ± 0.5	6.9 ± 0.9	20.7 ± 1.4	10.5 ± 1.8	14.0 ± 0.5	37.5 ± 1.8	40.0 ± 0.9	55.7 ± 1.4	59.3 ± 1.3	40.0 ± 1.5	35.7 ± 2.2	53.3 ± 1.1
**3k**	6.3 ± 0.5	19.2 ± 1.4	9.5 ± 0.8	17.2 ± 0.9	15.5 ± 0.7	10.5 ± 1.2	19.3 ± 0.7	35.0 ± 1.5	52.0 ± 1.8	78.7 ± 2.1	77.8 ± 2.6	50.0 ± 0.6	50.0 ± 1.7	56.7 ± 1.3
**3l**	6.3 ± 0.8	50.0 ± 3.0	4.8 ± 0.5	17.2 ± 0.9	20.7 ± 0.3	18.4 ± 0.5	14.0 ± 0.9	72.5 ± 3.2	36.0 ± 1.2	78.7 ± 2.7	80.2 ± 1.9	47.5 ± 1.5	50.0 ± 0.3	30.0 ± 0.9
**3m**	12.5 ± 0.5	30.8 ± 0.4	4.8 ± 0.3	20.7 ± 0.7	24.1 ± 0.9	10.5 ± 1.2	36.8 ± 1.0	47.5 ± 1.8	64.0 ± 3.3	77.0 ± 2.4	76.5 ± 2.0	55.0 ± 1.4	53.6 ± 1.6	63.3 ± 1.5
**3n**	12.5 ± 0.8	46.2 ± 0.7	14.3 ± 0.6	37.9 ± 0.8	20.7 ± 1.6	28.9 ± 1.7	14.0 ± 0.9	47.5 ± 1.2	60.0 ± 2.1	82.0 ± 0.6	80.2 ± 1.8	52.5 ± 2.2	57.1 ± 0.9	70.0 ± 1.3
**3o**	12.5 ± 0.7	50.0 ± 1.5	23.8 ± 1.0	27.6 ± 0.6	29.3 ± 0.9	10.5 ± 0.6	19.3 ± 1.3	42.5 ± 1.0	52.0 ± 2.3	63.9 ± 2.1	55.6 ± 2.5	35.0 ± 1.3	42.9 ± 2.2	46.7 ± 1.2
Carbendazim ^c^	<50	100 ± 0.0	100 ± 0.0	<50	100 ± 0.0	<50	100 ± 0.0	<50	<50	<50	100 ± 0.0	100 ± 0.0	100 ± 0.0	<50
chlorothalonil ^c^	73 ± 1	<50	86 ± 1	100 ± 0.0	<50	100 ± 0.0	100 ± 0.0	100 ± 0.0	73 ± 1	100 ± 0.0	100 ± 0.0	91 ± 1	91 ± 1	100 ± 0.0

^a^ Average of three replicates; All results are expressed as mean ± SD; Statistical analysis was conducted using SPSS 27.0 software. Statistical significance was defined as *p* < 0.05. ^b^ Abbreviations: AS—*Alternaria solani*; FG—*Fusarium graminearum*; PI—*Phytophthora infestans*; PC—*Phytophthora capsici*; SS—*Sclerotinia sclerotiorum*; BC—*Botrytis cinerea*; RS—*Rhizoctonia solani*; FC—*Fusarium oxysporum* f. sp. *cucumeris*; CH—*Cercospora arachidicola* Hori; PP—*Physalospora piricola*; RC—*Rhizoctonia cerealis*; BM—*Bipolaris maydis*; WA—watermelon anthracnose; FM—*Fusarium moniliforme*. ^c^ Commercial agricultural fungicides were used to compare antifungal activities.

**Table 5:** Predicted toxicity of compounds **3a**–**3o**.

Compounds	Acut Tox. Pred. (mg/kg) ^a^	Mutigenic Tox. Pred.	Carcinogenic Tox. Pred.
**3a**	75	negative	negative
**3b**	1000	negative	negative
**3c**	75	negative	positive
**3d**	75	negative	positive
**3e**	75	negative	negative
**3f**	75	negative	positive
**3g**	1000	negative	positive
**3h**	75	negative	negative
**3i**	75	negative	negative
**3j**	1000	positive	positive
**3k**	1000	positive	positive
**3l**	200	negative	negative
**3m**	75	negative	negative
**3n**	200	negative	positive
**3o**	75	negative	negative

^a^ Rat, oral, LD_50_.

## Data Availability

The original contributions presented in this study are included in the article/Supplementary Material. Further inquiries can be directed to the corresponding authors.

## Supplementary Materials

The following supporting information can be downloaded at: https://www.mdpi.com/article/10.3390/ijms27010260/s1.
