# Supplementary material for: Discovery of Triazone Derivatives Containing Acylhydrazone and Phenoxypyridine Motifs as Novel Insecticidal and Antiphytopathogenic Fungus Agents
Source: Int J Mol Sci. 2025 Dec 26;27(1):260. doi: 10.3390/ijms27010260 (PMC12785405; doi:10.3390/ijms27010260)
Supplement: Supplementary file 1 [file ijms-27-00260-s001.zip › ijms-4016496-supplementary.pdf]

Supporting Information

Synthesis and Insecticidal/Fungicidal Activities of Triazone Derivatives Containing Acylhydrazone and Phenoxypyridine Moieties

Peipei Cui[1], Yan Yang[2*]

[1] College of Architecture and Arts, Taiyuan University of Technology, Jinzhong 030060, China;

[2] College of Chemistry and Chemical Engineering, Taiyuan University of Technology, Taiyuan 030024, China

* Correspondence: yy529222@163.com; Tel.: +86-0351-6010111

General Procedures for the Preparation of Compounds **3a-3o** ……….…….…………….………… S2

^{1}H NMR, ^{13}C NMR and HRMS Data for Compounds **3a-3o**………….………………………S2–S7

^{1}H NMR and ^{13}C NMR spectrum for Compounds **3a-3o**…………….………….…………...S8–S23

Biological assay…………………………………………………………......…...…..……S24–S25

General Procedures for the Preparation of Compounds 3a-3o.

To a solution of compound **1** (0.51 g, 4.00 mmol) and compounds **2a-2o** (0.85 g, 4.00 mmol) in methanol (80 mL) was added *p*-toluene sulfonic acid (0.14 g, 0.80 mmol), and then the mixture was refluxed for 8 h. The solution was cooled and then concentrated under reduced pressure. The crude product was purified by flash chromatography on silica gel using petroleum ether (60-90 ℃) and ethyl acetate (v/v = 5:1) as eluent to give the target compounds **3a-3o**.

Data for (E)-N-(6-methyl-3-oxo-2,3-dihydro-1,2,4-triazin-4(5H)-yl)-2-(1-(6-(o-tolyloxy) pyridin-3-yl)ethylidene)hydrazinecarboxamide (3a). Yellow solid, m.p. 218-219 °C, yield 54%; ^{1}H NMR (400 MHz, DMSO-*d*$_6$): δ 9.96 (s, 1H), 9.77 (s, 1H), 9.22 (s, 1H), 8.56 (s, 1H), 8.45 (d, J = 8.0 Hz, 1H), 7.31 (d, J = 7.2 Hz, 1H), 7.26-7.22 (m, 1H), 7.17-7.14 (m, 1H), 7.06 (d, J = 8.0 Hz, 1H), 7.02 (d, J = 8.8 Hz, 1H), 4.10 (s, 2H), 2.18 (s, 3H), 2.08 (s, 3H), 1.86 (s, 3H); ^{13}C NMR (100 MHz,DMSO-*d*$_6$): δ 163.2, 155.1, 151.9, 151.6, 145.6, 144.7, 143.5, 137.9, 131.2, 130.2, 128.7, 127.2, 125.1, 122.0, 110.0, 52.1, 19.8, 16.0, 13.0; ESI-HRMS (m/z): Calcd. for $C_{19}H_{22}N_7O_3$ $[M+H]^+$ 396.1779, found 396.1779.

Data for (E)-2-(1-(6-(2-ethoxyphenoxy)pyridin-3-yl)ethylidene)-N-(6-methyl-3-oxo-2,3-dihydro-1,2,4-triazin-4(5H)-yl)hydrazinecarboxamide (3b). White solid, m.p. 217-218 ℃, yield 61%; ^{1}H NMR (400 MHz, DMSO-*d*$_6$): δ 9.94 (s, 1H), 9.77 (s, 1H), 9.22 (s, 1H), 8.54 (s, 1H), 8.43 (d, J = 8.4 Hz, 1H), 7.21-7.18 (m, 1H), 7.15-7.10 (m, 2H), 6.99 (s, 1H), 6.97 (s, 1H), 4.10 (s, 2H), 3.94 (q, J = 6.8 Hz, 2H), 2.18 (s, 3H),1.87 (s, 3H), 1.05 (t, J = 6.8 Hz, 3H); ^{13}C NMR (100 MHz, DMSO-*d*$_6$): δ 163.5, 155.1,151.7, 150.7,

145.3, 144.8, 143.6, 142.4, 137.5, 128.5, 126.0, 123.0, 120.8, 114.2, 109.7, 63.7, 52.1, 19.9, 14.5, 13.0; ESI-HRMS (m/z): Calcd. for $C_{20}H_{24}N_7O_4$ $[M+H]^+$ 426.1884, found 426.1887.

Data for (E)-2-(1-(6-(4-ethylphenoxy)pyridin-3-yl)ethylidene)-N-(6-methyl-3-oxo-2,3-dihydro-1,2,4-triazin-4(5H)-yl)hydrazinecarboxamide (3c). Yellow solid, m.p. 214-215 °C, yield 85%; ^{1}H NMR (400 MHz, DMSO-d_6): δ 9.95(s, 1H), 9.76 (s, 1H), 9.22 (s, 1H), 8.59 (s, 1H), 8.45 (d, J = 8.4 Hz, 1H), 7.25 (d, J = 8.4 Hz, 2H), 7.05 (d, J = 8.4 Hz, 2H), 7.00 (d, J = 8.4 Hz, 1H), 4.11 (s, 2H), 2.62 (q, J = 7.6 Hz, 2H), 2.19 (s, 3H),1.87 (s, 3H), 1.20 (t, J = 7.6 Hz, 3H); ^{13}C NMR (100 MHz, DMSO-d_6): δ 163.4, 155.1, 151.6, 151.5, 145.6, 144.8, 143.5, 140.1, 137.8, 128.9, 128.8, 121.2, 110.6, 52.1, 27.6, 19.9, 15.8, 13.0; ESI-HRMS (m/z): Calcd. for $C_{20}H_{24}N_7O_3$ $[M+H]^+$410.1935, found 410.1941.

Data for (E)-2-(1-(6-(4-isopropylphenoxy)pyridin-3-yl)ethylidene)-N-(6-methyl-3-oxo-2,3-dihydro-1,2,4-triazin-4(5H)-yl)hydrazinecarboxamide (3d). Yellow solid, m.p. 222-223 °C, yield 87%; ^{1}H NMR (400 MHz, DMSO-d_6): δ 9.96(s, 1H), 9.77 (s, 1H), 9.23 (s, 1H), 8.59 (s, 1H), 8.45 (d, J = 8.4 Hz, 1H), 7.28 (d, J = 7.6 Hz, 2H), 7.06 (d, J = 7.2 Hz, 2H), 7.01 (d, J = 8.4 Hz, 1H), 4.11 (s, 2H), 2.91 (m, 1H), 2.19 (s, 3H), 1.87 (s, 3H), 1.22 (d, J = 6.4 Hz, 6H); ^{13}C NMR (100 MHz, DMSO-d_6): δ 163.4, 155.1, 151.6, 145.6, 144.7, 143.4, 137.8, 128.9, 127.4, 121.1, 110.6, 52.1, 32.9, 24.0, 19.9, 13.0; ESI-HRMS (m/z): Calcd. for $C_{21}H_{26}N_7O_3$ $[M+H]^+$424.2092, found 424.2096.

Data for (E)-2-(1-(6-(4-tert-butylphenoxy)pyridin-3-yl)ethylidene)-N-(6-methyl-3-oxo-2,3- dihydro-1,2,4-triazin-4(5H)-yl)hydrazinecarboxamide (3e). White solid, m.p.

208-209 °C, yield 88%; ^{1}H NMR (400 MHz, DMSO-d_6): δ 9.96 (s, 1H), 9.76 (s, 1H), 9.22 (s, 1H), 8.59 (s, 1H), 8.46 (d, J = 8.8 Hz, 1H), 7.43 (d, J = 8.4 Hz, 2H), 7.06 (d, J = 8.4 Hz, 2H), 7.02 (d, J = 8.8 Hz, 1H), 4.11 (s, 2H), 2.19 (s, 3H),1.87 (s, 3H), 1.30 (s, 9H); ^{13}C NMR (100 MHz, DMSO-d_6): δ 163.4, 155.1, 151.6, 151.3, 147.0, 145.6, 144.7, 143.4, 137.8, 128.9, 126.3, 120.8, 110.6, 52.1, 34.2, 31.3, 19.9, 13.0; ESI-HRMS (m/z): Calcd. for $C_{22}H_{28}N_7O_3$ [M+H]$^+$ 438.2248; found 438.2253.

Data for (E)-2-(1-(6-(4-(benzyloxy)phenoxy)pyridin-3-yl)ethylidene)-N-(6-methyl-3-oxo-2,3-dihydro-1,2,4-triazin-4(5H)-yl)hydrazinecarboxamide (3f). Yellow solid, m.p. 212-213 °C, yield 32%; ^{1}H NMR (400 MHz, DMSO-d_6): δ 9.95(s, 1H), 9.77 (s, 1H), 9.22 (s, 1H), 8.58 (s, 1H), 8.44 (d, J = 8.4 Hz, 1H), 7.47 (d, J = 8.0 Hz, 2H), 7.43-7.39 (m, 2H), 7.35 (d, J = 6.4 Hz, 1H), 7.10-7.03 (m, 4H), 6.98 (d, J = 8.4 Hz, 1H), 5.12 (s, 2H), 4.11 (s, 2H), 2.19 (s, 3H),1.87 (s, 3H); ^{13}C NMR (100 MHz, DMSO-d_6): δ 163.6, 155.3, 155.1, 151.6, 147.1, 145.5, 144.8, 143.5, 137.8, 137.1, 128.8, 128.5, 127.9, 127.7, 122.5, 115.6, 110.3, 69.6, 52.1, 19.9, 13.0; ESI-HRMS (m/z): Calcd. for $C_{25}H_{26}N_7O_4$ [M+H]$^+$ 488.2041, found 488.2049.

Data for (E)-2-(1-(6-(4-benzoylphenoxy)pyridin-3-yl)ethylidene)-N-(6-methyl-3-oxo-2,3-dihydro-1,2,4-triazin-4(5H)-yl)hydrazinecarboxamide (3g). White solid, m.p. 234-235 °C, yield 22%; ^{1}H NMR (400 MHz, DMSO-d_6): δ 10.00 (s, 1H),9.77(s, 1H), 9.27 (s, 1H), 8.69 (s, 1H), 8.53 (d, J = 8.4 Hz, 1H), 7.82 (d, J = 8.0 Hz, 2H), 7.75 (d, J = 7.2 Hz, 2H), 7.70-7.67 (m, 1H), 7.60-7.56 (m, 2H), 7.32 (d, J = 8.4 Hz, 2H), 7.18 (d, J = 8.4 Hz, 1H), 4.11 (s, 2H), 2.22 (s, 3H), 1.87 (s, 3H); ^{13}C NMR (100 MHz, DMSO-d_6): δ 194.7, 162.3, 157.6, 155.1, 151.6, 145.7, 144.8, 143.3, 138.3, 137.2, 133.0, 132.6, 131.8,

129.9, 129.5, 128.6, 120.7, 111.6, 52.1, 19.9, 13.0; ESI-HRMS (m/z): Calcd. for $C_{25}H_{24}N_7O_4$ [M+H]$^+$ 486.1884, found 486.1891.

Data for (E)-2-(1-(6-(4-bromophenoxy)pyridin-3-yl)ethylidene)-N-(6-methyl-3-oxo-2,3-dihydro-1,2,4-triazin-4(5H)-yl)hydrazinecarboxamide (3h). Yellow solid, m.p. 238-239 °C, yield 75%; ^{1}H NMR (400 MHz, DMSO-d_6): δ 9.98 (s, 1H), 9.77 (s, 1H), 9.25 (s, 1H), 8.61 (s, 1H), 8.49 (d, J = 8.8 Hz, 1H), 7.60 (d, J = 8.4 Hz, 2H), 7.14 (d, J = 8.4 Hz, 2H), 7.09 (d, J = 8.8 Hz, 1H), 4.11 (s, 2H), 2.20 (s, 3H),1.87 (s, 3H); ^{13}C NMR (100 MHz, MSO-d_6): δ 162.7, 155.1, 153.0, 151.6, 145.5, 144.7, 143.3, 138.1, 132.4, 129.4, 123.6, 116.6, 111.0, 52.1, 19.8, 13.0; ESI-HRMS (m/z): Calcd. for $C_{18}H_{19}BrN_7O_3$ [M+H]$^+$ 460.0727, found 460.0722.

Data for (E)-2-(1-(6-(4-iodophenoxy)pyridin-3-yl)ethylidene)-N-(6-methyl-3-oxo-2,3-dihydro-1,2,4-triazin-4(5H)-yl)hydrazinecarboxamide (3i). White solid, m.p. 244-245 °C, yield 72%; ^{1}H NMR (400 MHz, DMSO-d_6): δ 9.97(s, 1H), 9.77 (s, 1H), 9.24 (s, 1H), 8.61 (s, 1H), 8.48 (d, J = 8.8 Hz, 1H), 7.75 (d, J = 8.4 Hz, 2H), 7.08 (d, J = 8.8 Hz, 1H), 6.99 (d, J = 8.4 Hz, 2H), 4.11 (s, 2H), 2.19 (s, 3H),1.87 (s, 3H); ^{13}C NMR (100 MHz, DMSO-d_6): δ 162.7, 155.1, 153.7, 151.6, 145.5, 144.8, 143.3, 138.3, 138.1, 129.4, 123.8, 111.0, 88.8, 52.1, 19.9, 13.0; ESI-HRMS (m/z): Calcd. for $C_{18}H_{19}IN_7O_3$ [M+H]$^+$ 508.0589, found 508.0590.

Data for (E)-N-(6-methyl-3-oxo-2,3-dihydro-1,2,4-triazin-4(5H)-yl)-2-(1-(6-(3-nitrophenoxy)pyridin-3-yl)ethylidene)hydrazinecarboxamide (3j). Yellow solid, m.p. 261-262 °C, yield 81%; ^{1}H NMR (400 MHz, DMSO-d_6): δ 10.00 (s, 1H, NH), 9.77 (s, 1H), 9.27 (s, 1H), 8.65 (s, 1H), 8.54 (d, J = 6.8 Hz, 1H), 8.10 (d, J = 7.2 Hz, 1H), 8.03 (s,

1H), 7.75-7.69 (m, 2H), 7.19 (d, *J* = 8.4 Hz, 1H), 4.11 (s, 2H), 2.21 (s, 3H),1.87 (s, 3H); ^{13}C NMR (100 MHz, DMSO-*d*$_6$): δ 162.2, 155.0, 154.0, 151.6, 148.4, 145.4, 144.7, 143.2, 138.3, 130.9, 129.8, 128.2, 119.5, 116.2, 111.2, 52.1, 19.8, 12.9; ESI-HRMS (m/z): Calcd. for $C_{18}H_{19}N_8O_5$ [M+H]$^+$ 427.1473, found 427.1474.

Data for (E)-2-(1-(6-(3-aminophenoxy)pyridin-3-yl)ethylidene)-N-(6-methyl-3-oxo-2,3-dihydro-1,2,4-triazin-4(5H)-yl)hydrazinecarboxamide (3k). Yellow solid, m.p. 233-234 °C, yield 80%; ^{1}H NMR (400 MHz, DMSO-*d*$_6$): δ 9.95 (s, 1H), 9.76 (s, 1H), 9.22 (s, 1H), 8.63 (s, 1H), 8.42 (d, *J* = 8.4 Hz, 1H), 7.02 (t, *J* = 8.0 Hz, 1H), 6.93 (d, *J* = 8.4 Hz, 1H), 6.39 (d, *J* = 8.0 Hz, 1H), 6.27 (s, 1H), 6.22 (d, *J* = 7.6 Hz, 1H), 5.24 (s, 2H), 4.11 (s, 2H), 2.20 (s, 3H),1.87 (s, 3H); ^{13}C NMR (100 MHz, DMSO-*d*$_6$): δ 163.4, 155.1, 155.0, 151.6, 150.3, 145.8, 144.8, 143.5, 137.7, 129.8, 128.9, 110.6, 110.4, 107.8, 106.1, 52.1, 19.9, 13.0; ESI-HRMS (m/z): Calcd. for $C_{18}H_{21}N_8O_3$ [M+H]$^+$ 397.1731, found 397.1739.

Data for (E)-2-(1-(6-(4-isopropyl-3-methylphenoxy)pyridin-3-yl)ethylidene)-N-(6-methyl-3-oxo-2,3-dihydro-1,2,4-triazin-4(5H)-yl)hydrazinecarboxamide (3l). White solid, m.p. 224-225 °C, yield 78%; ^{1}H NMR (400 MHz, DMSO-*d*$_6$): δ 9.95 (s, 1H), 9.76 (s, 1H), 9.22 (s, 1H), 8.60 (s, 1H), 8.44 (d, *J* = 8.4 Hz, 1H), 7.26(d, *J* = 8.0 Hz, 1H), 6.99 (d, *J* = 8.8 Hz, 1H), 6.93-6.91 (m, 2H), 4.11 (s, 2H), 3.10 (m, 1H), 2.29 (s, 3H), 2.19 (s, 3H), 1.87 (s, 3H), 1.19 (d, *J* = 6.4 Hz, 6H); ^{13}C NMR (100 MHz, DMSO-*d*$_6$): δ 163.5, 155.1, 151.6, 151.2, 145.6, 144.7, 143.5, 142.7, 137.8, 136.2, 128.8, 125.7, 122.6, 118.8, 110.5, 52.1, 28.3, 23.2, 19.8, 18.9, 13.0; ESI-HRMS (m/z): Calcd. for $C_{22}H_{28}N_7O_3$ [M+H]$^+$ 438.2248, found 438.2254.

Data for (E)-2-(1-(6-(2-isopropyl-5-methylphenoxy)pyridin-3-yl)ethylidene)-N-(6-methyl-3-oxo-2,3-dihydro-1,2,4-triazin-4(5H)-yl)hydrazinecarboxamide (3m). White solid, m.p. 236-237 °C, yield 25%; ^{1}H NMR (400 MHz, DMSO-d_6): δ 9.94 (s, 1H), 9.76 (s, 1H), 9.21 (s, 1H), 8.58 (s, 1H), 8.43 (d, J = 8.8 Hz, 1H), 7.26(d, J = 8.0 Hz, 1H), 7.03 (d, J = 8.0 Hz, 1H), 6.99 (d, J =8.8 Hz, 1H), 6.83(s, 1H), 4.11 (s, 2H), 3.00-2.94 (m, 1H), 2.26 (s, 3H), 2.19 (s, 3H), 1.87 (s, 3H), 1.10 (d, J = 7.2 Hz, 6H); ^{13}C NMR (100 MHz, DMSO-d_6): δ 163.8, 155.1, 151.6, 150.6, 145.7, 144.8, 143.5, 137.9, 137.1, 136.3, 128.7, 126.6, 126.1, 122.6, 110.0, 52.1, 26.5, 23.0, 20.4, 19.9, 13.0; ESI-HRMS (m/z): Calcd. for $C_{22}H_{28}N_7O_3$ $[M+H]^+$ 438.2248, found 438.2246.

Data for (E)-2-(1-(6-(4-allyl-2-methoxyphenoxy)pyridin-3-yl)ethylidene)-N-(6-methyl-3-oxo-2,3-dihydro-1,2,4-triazin-4(5H)-yl)hydrazinecarboxamide (3n). White solid, m.p. 241-242 °C, yield 61%; ^{1}H NMR (400 MHz, DMSO-d_6): δ 9.93 (s, 1H), 9.76 (s, 1H), 9.20 (s, 1H), 8.52 (s, 1H), 8.40 (d, J = 8.8 Hz, 1H), 7.05 (d, J = 7.6 Hz, 1H), 6.96-6.94 (m, 2H), 6.79 (d, J = 7.6 Hz, 1H), 6.03-5.97 (m, 1H), 5.15-5.06 (m, 1H), 4.10 (s, 2H), 3.66 (s, 3H), 3.39(d, J = 5.6 Hz, 2H), 2.17 (s, 3H), 1.87 (s, 3H); ^{13}C NMR (100 MHz, DMSO-d_6): δ 163.4, 155.1, 151.7, 151.3, 145.4, 144.8, 143.6, 140.0, 138.0, 137.7, 137.6, 128.5, 122.9, 120.5, 116.0, 113.1, 109.6, 55.5, 52.1, 19.9, 13.0; ESI-HRMS (m/z): Calcd. for $C_{22}H_{26}N_7O_4$ $[M+H]^+$ 452.2041, found 452.2044.

Data for (E)-2-(1-(6-(2-benzoyl-4-methoxyphenoxy)pyridin-3-yl)ethylidene)-N-(6-methyl-3-oxo-2,3-dihydro-1,2,4-triazin-4(5H)-yl)hydrazinecarboxamide (3o). White solid, m.p. 210-211 °C, yield 49%; ^{1}H NMR (400 MHz, DMSO-d_6): δ 9.92 (s, 1H), 9.76 (s, 1H), 9.19 (s, 1H), 8.47 (s, 1H), 8.29 (d, J = 8.8 Hz, 1H), 7.58-7.53 (m, 4H), 7.41-7.38

(m, 2H), 6.98 (d, *J* = 8.4 Hz, 1H), 6.87 (s, 1H), 6.57 (d, *J* = 8.8 Hz, 1H), 4.10 (s, 2H), 3.85 (s, 3H), 2.14 (s, 3H), 1.87 (s, 3H); ^{13}C NMR (100 MHz, DMSO-d_6): δ 193.6, 162.8, 162.6, 155.1, 153.0, 151.6, 145.0, 144.8, 143.3, 137.8, 137.7, 132.7, 132.0, 129.0, 128.9, 128.2, 124.2, 110.9, 110.2, 108.6, 55.8, 52.1, 19.9, 12.9; ESI-HRMS (m/z): Calcd. for $C_{26}H_{26}N_7O_5$ $[M+H]^+$ 516.1990, found 516.1986.

^{1}H NMR and ^{13}C NMR spectrum for compounds 3a-3o:

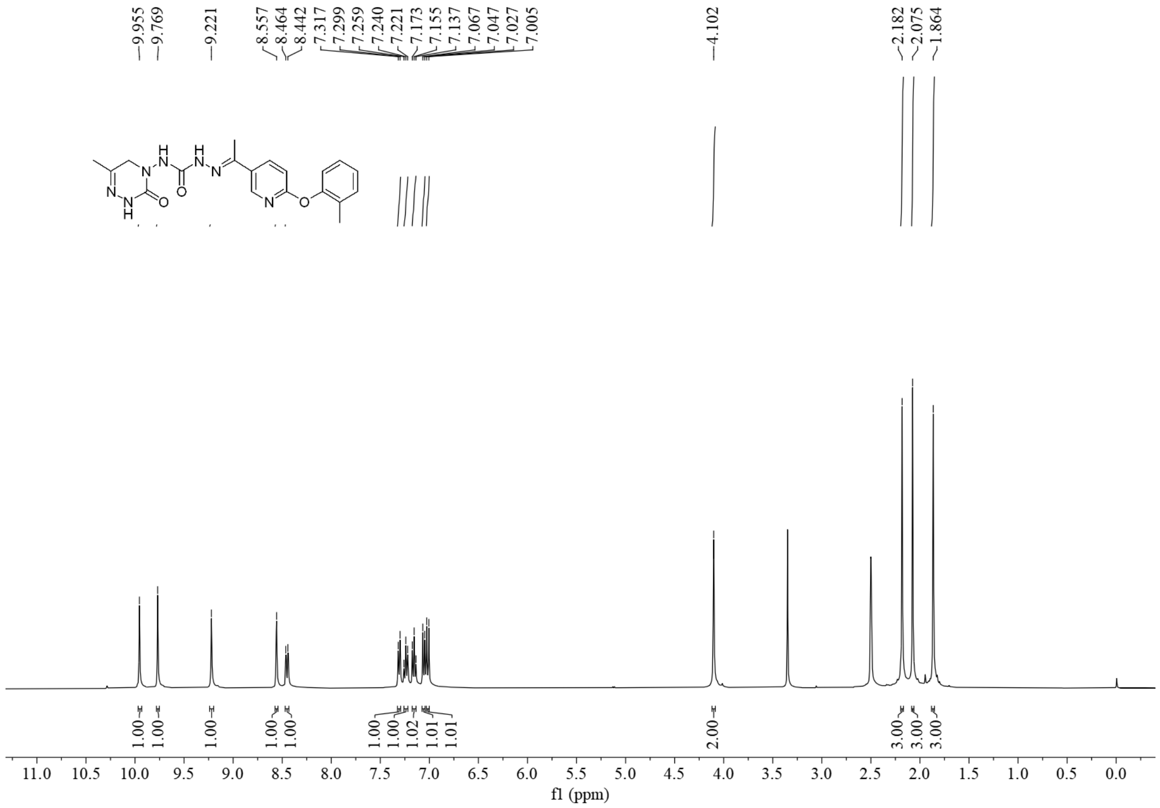

Figure S1 ^{1}H NMR spectrum of **3a**

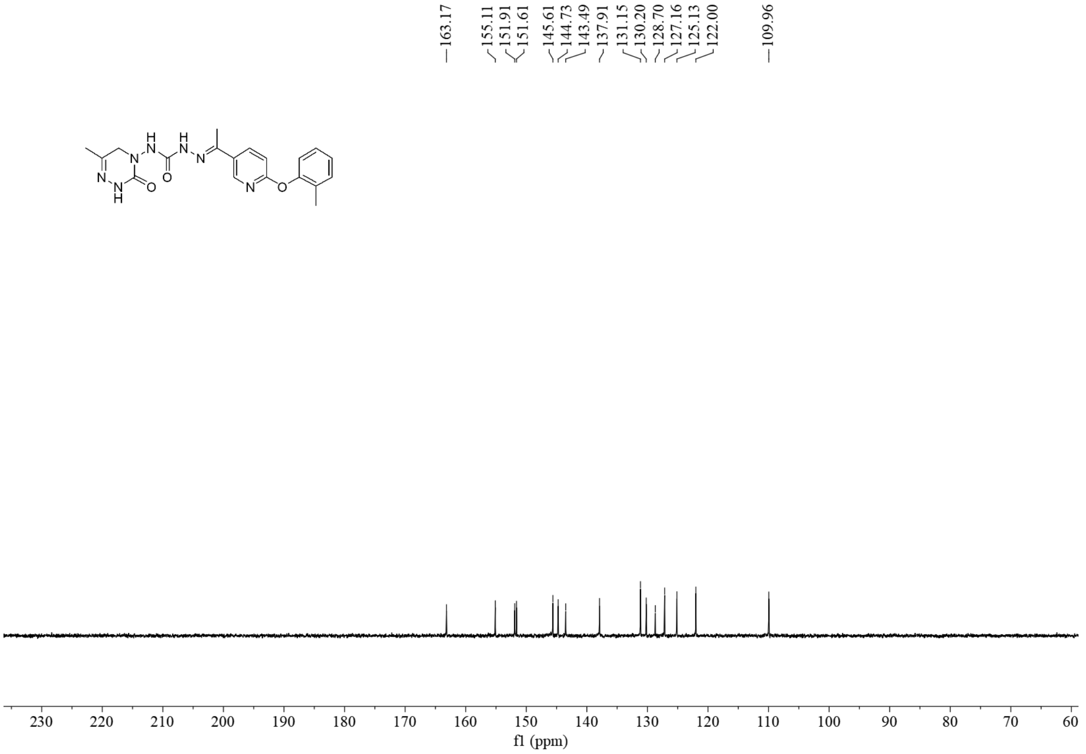

Figure S2 ^{13}C NMR spectrum of **3a**

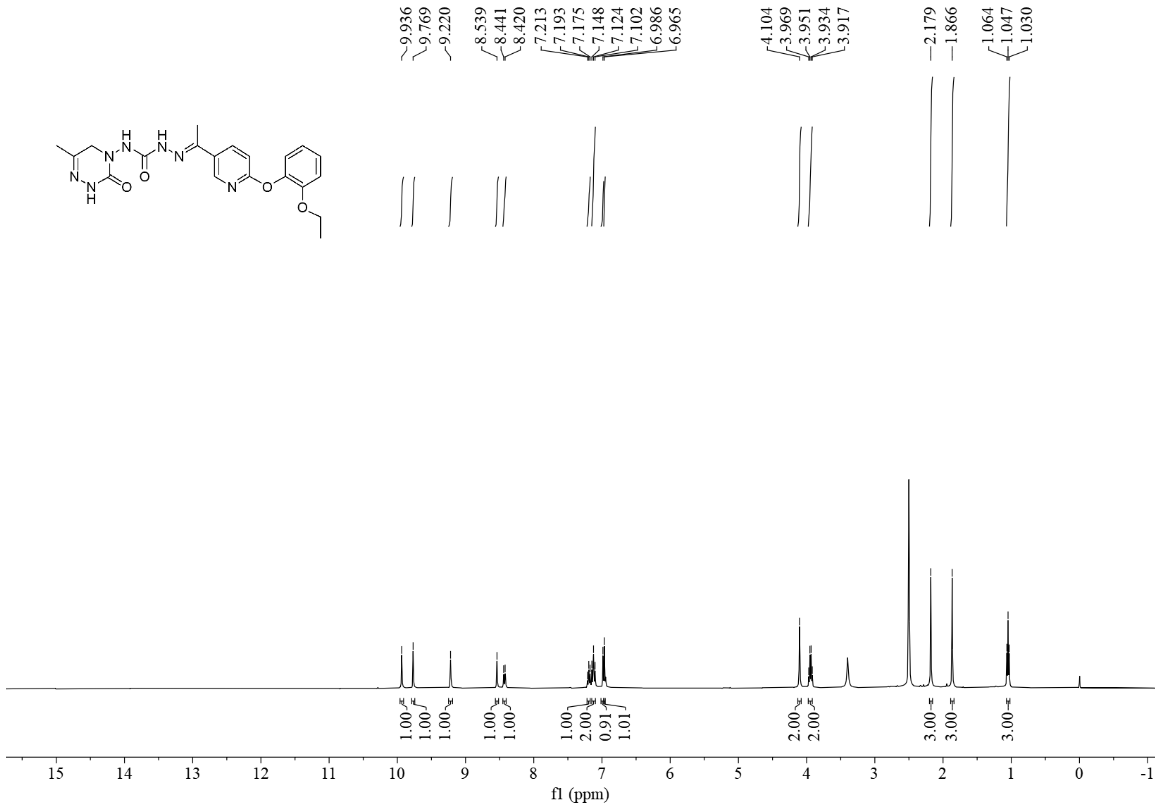

Figure S3 ^{1}H NMR spectrum of **3b**

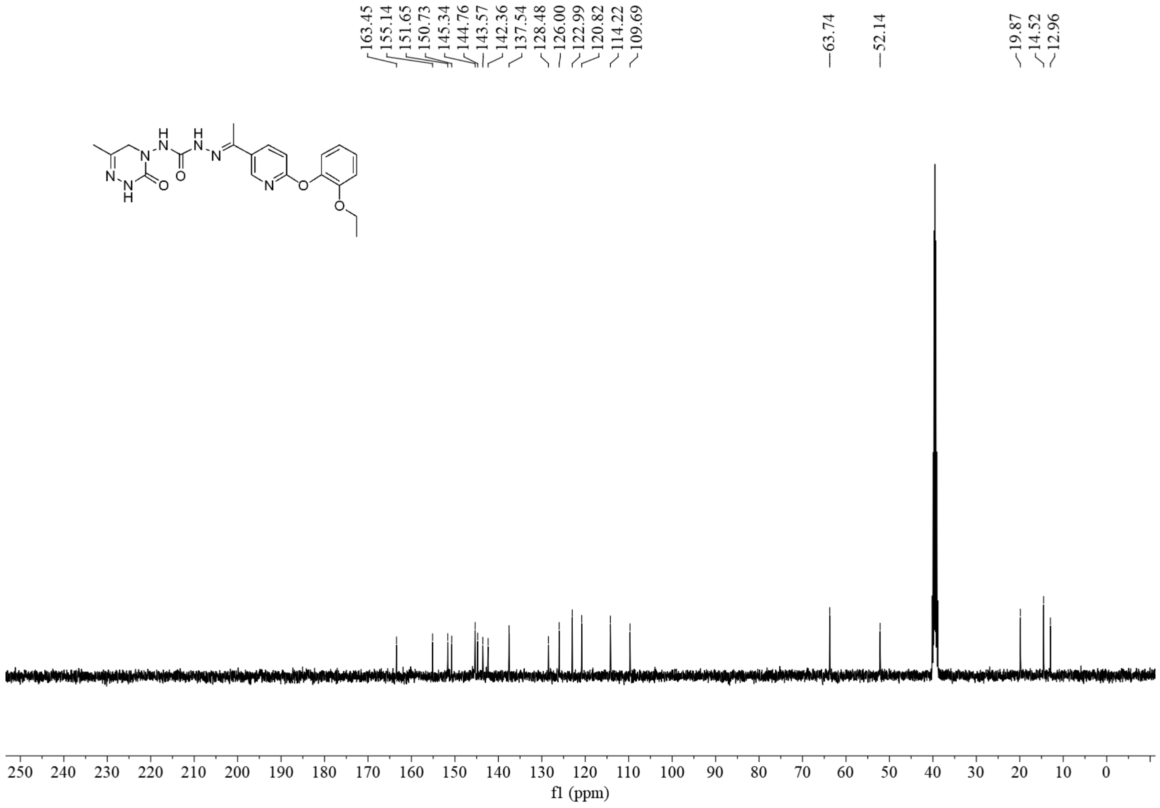

Figure S4 ^{13}C NMR spectrum of **3b**

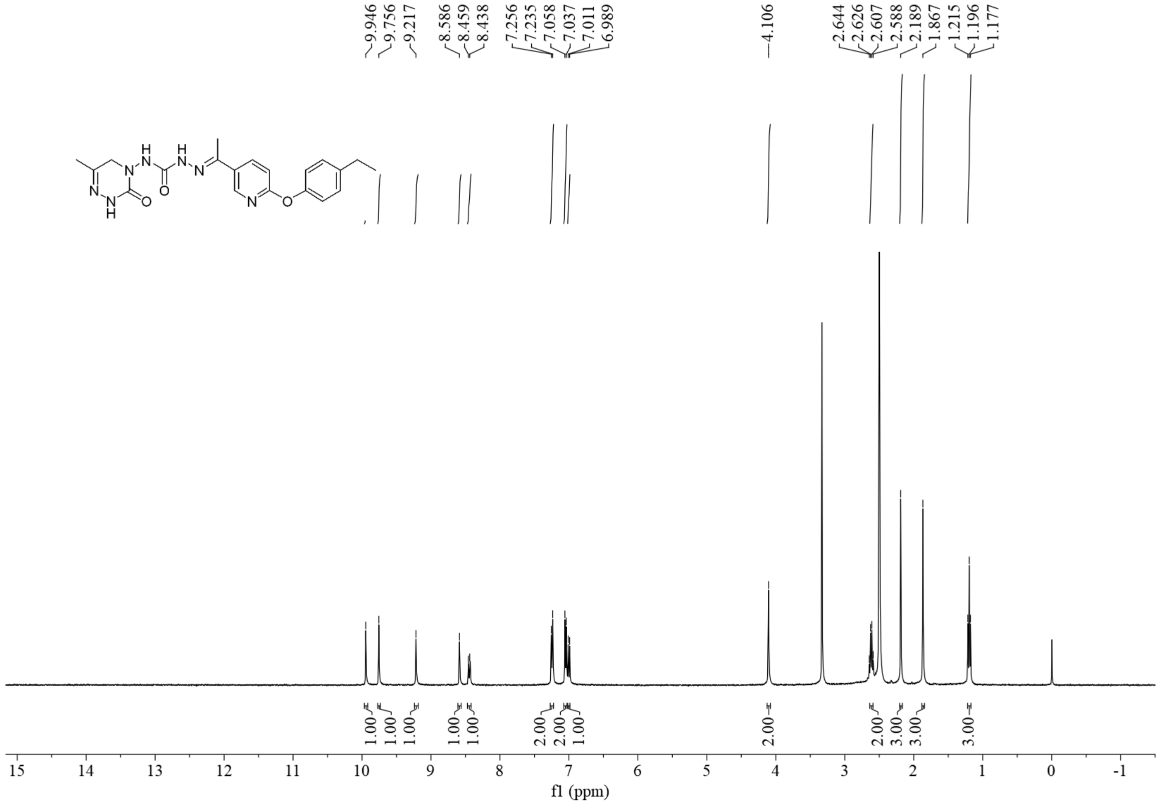

Figure S5 ^{1}H NMR spectrum of **3c**

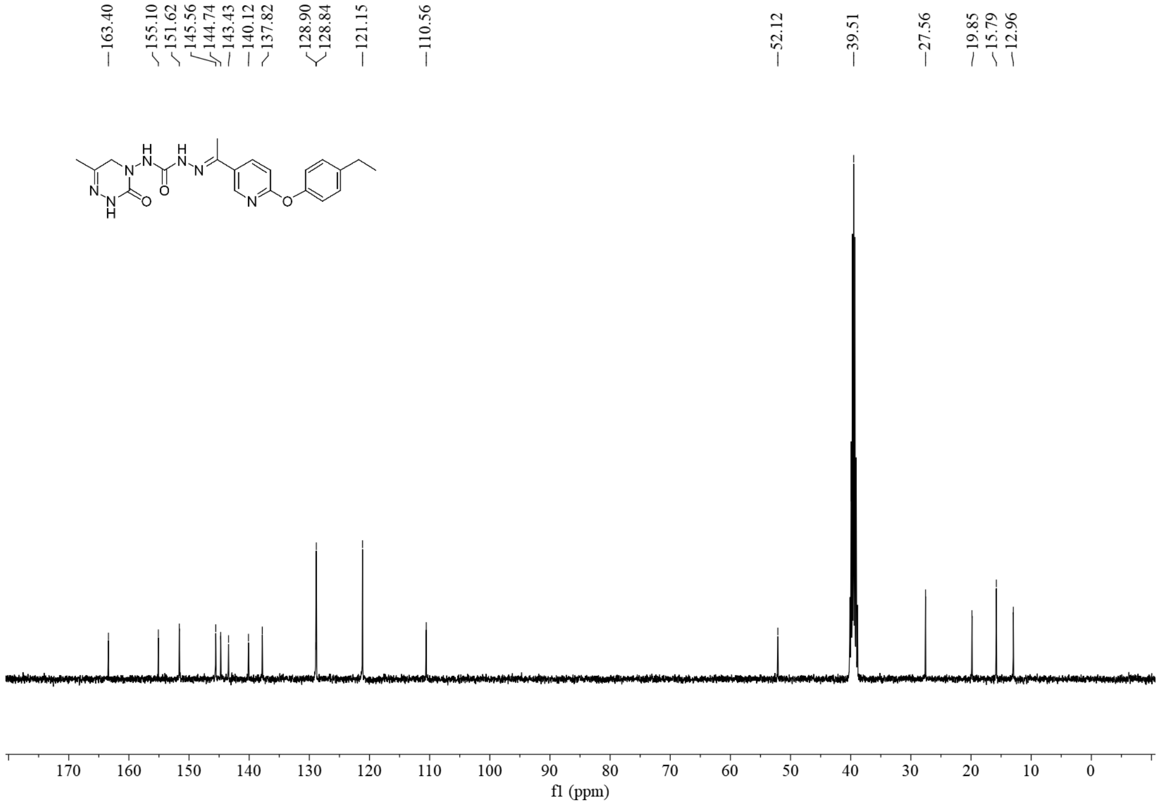

Figure S6 ^{13}C NMR spectrum of **3c**

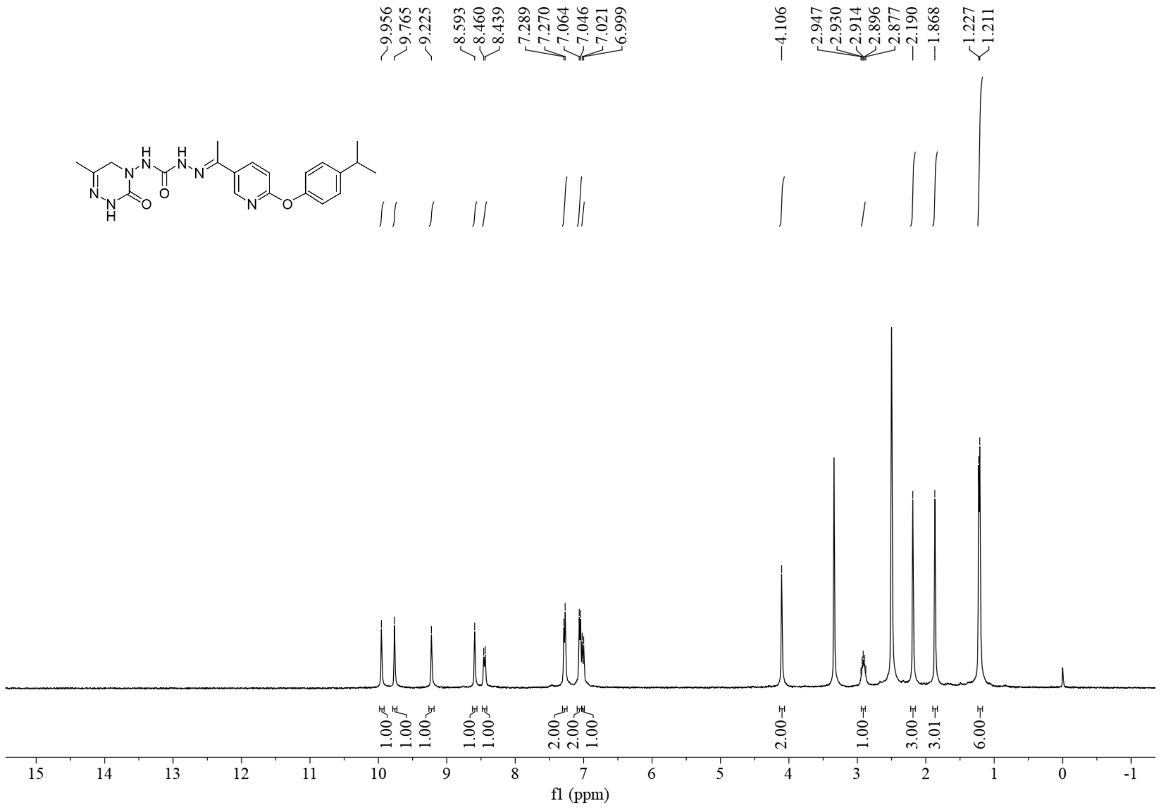

Figure S7 ^{1}H NMR spectrum of **3d**

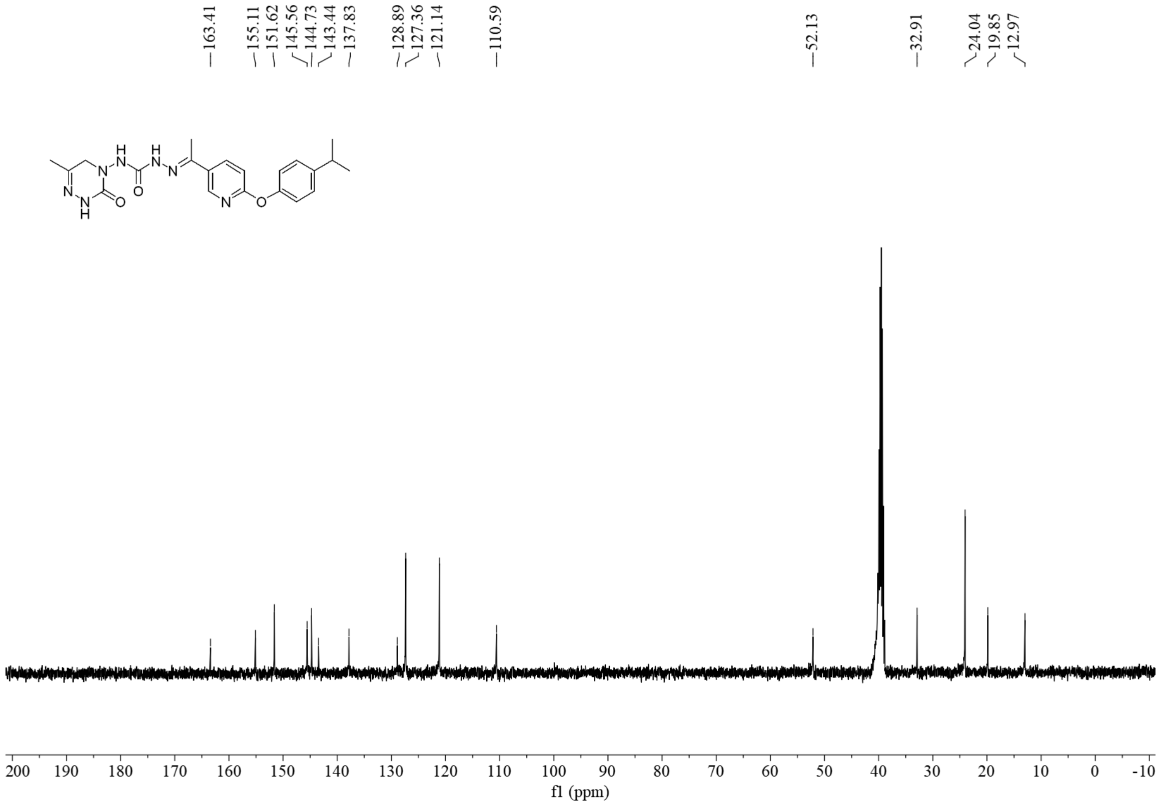

Figure S8 ^{13}C NMR spectrum of **3d**

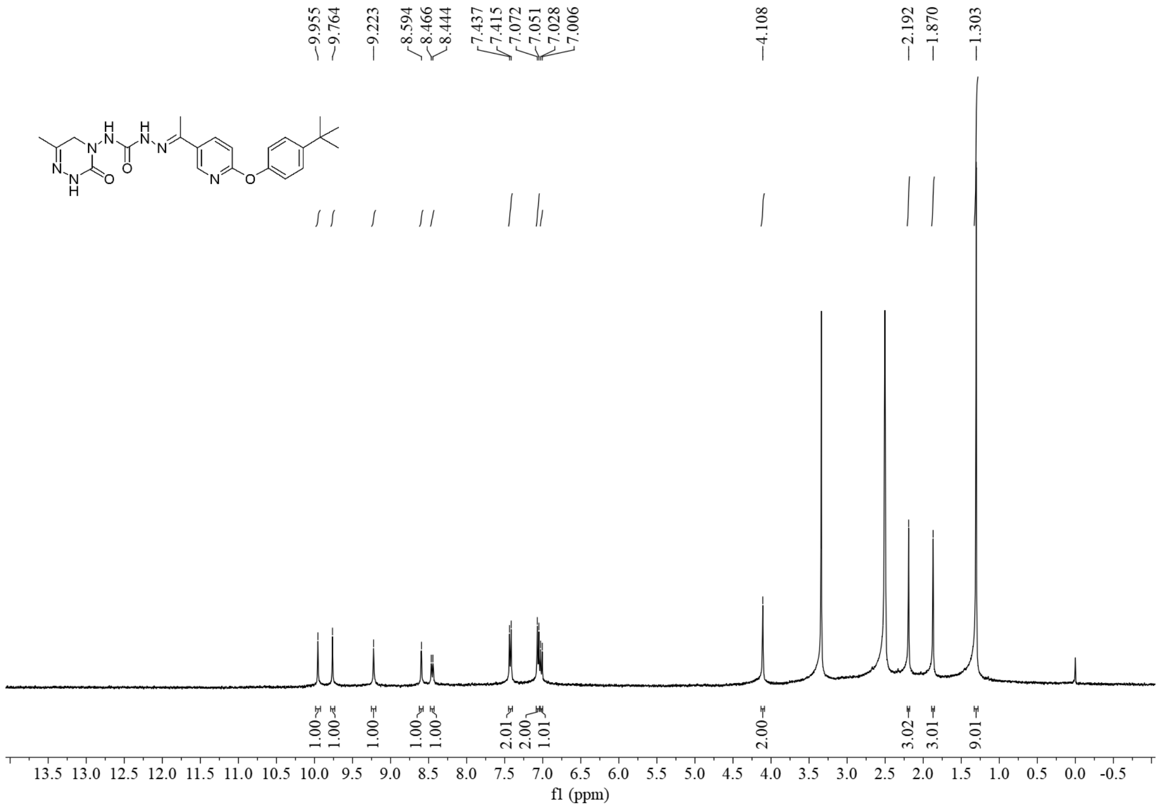

Figure S9 ^{1}H NMR spectrum of **3e**

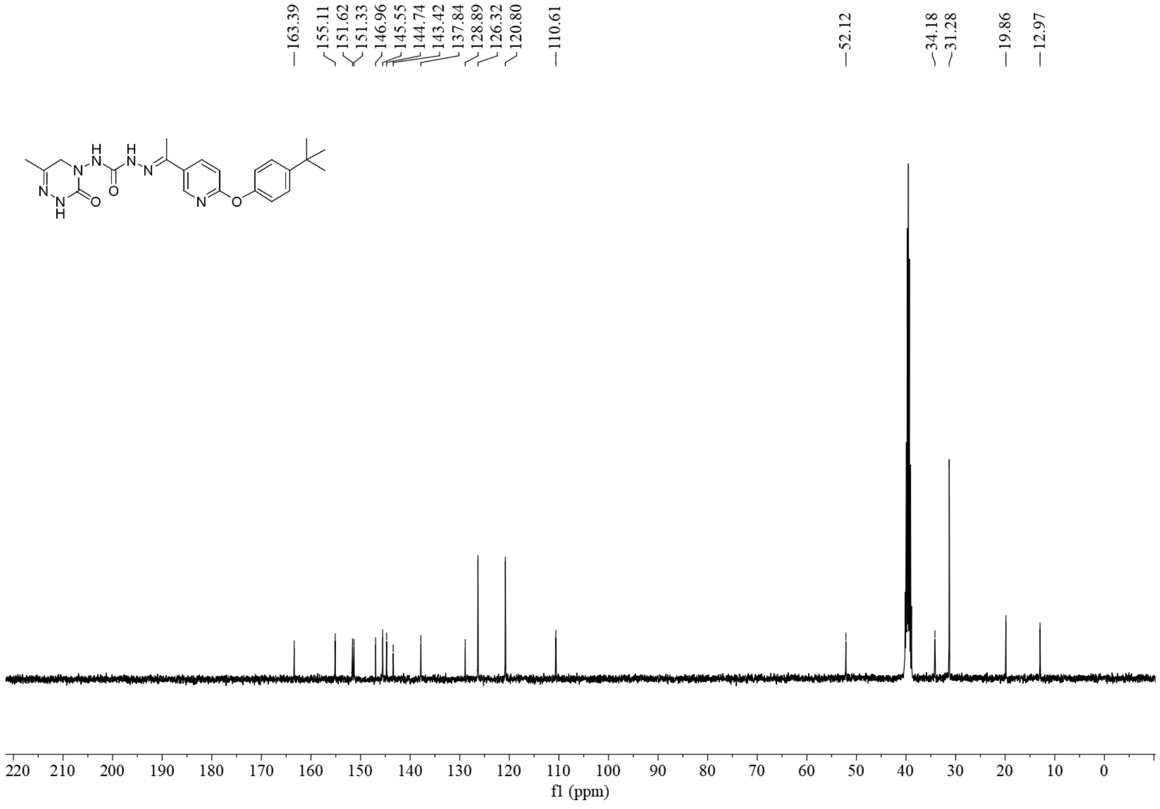

Figure S10 ^{13}C NMR spectrum of **3e**

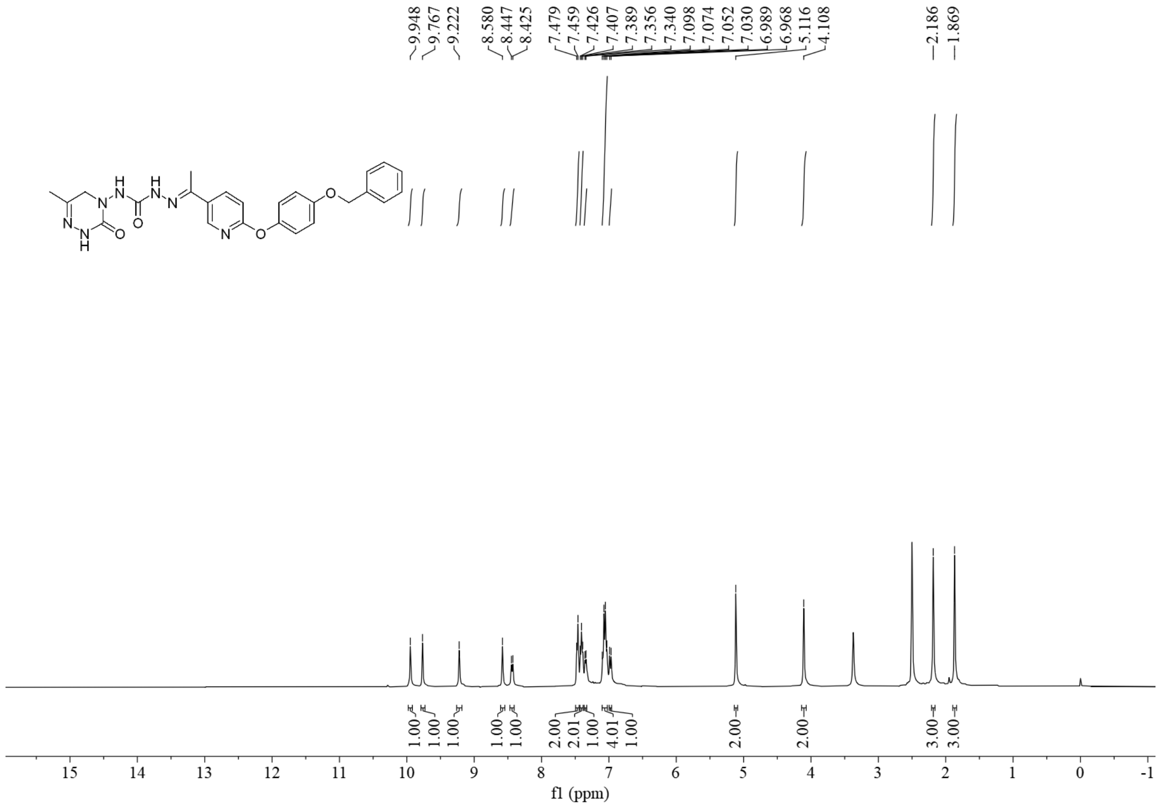

Figure S11 ^{1}H NMR spectrum of **3f**

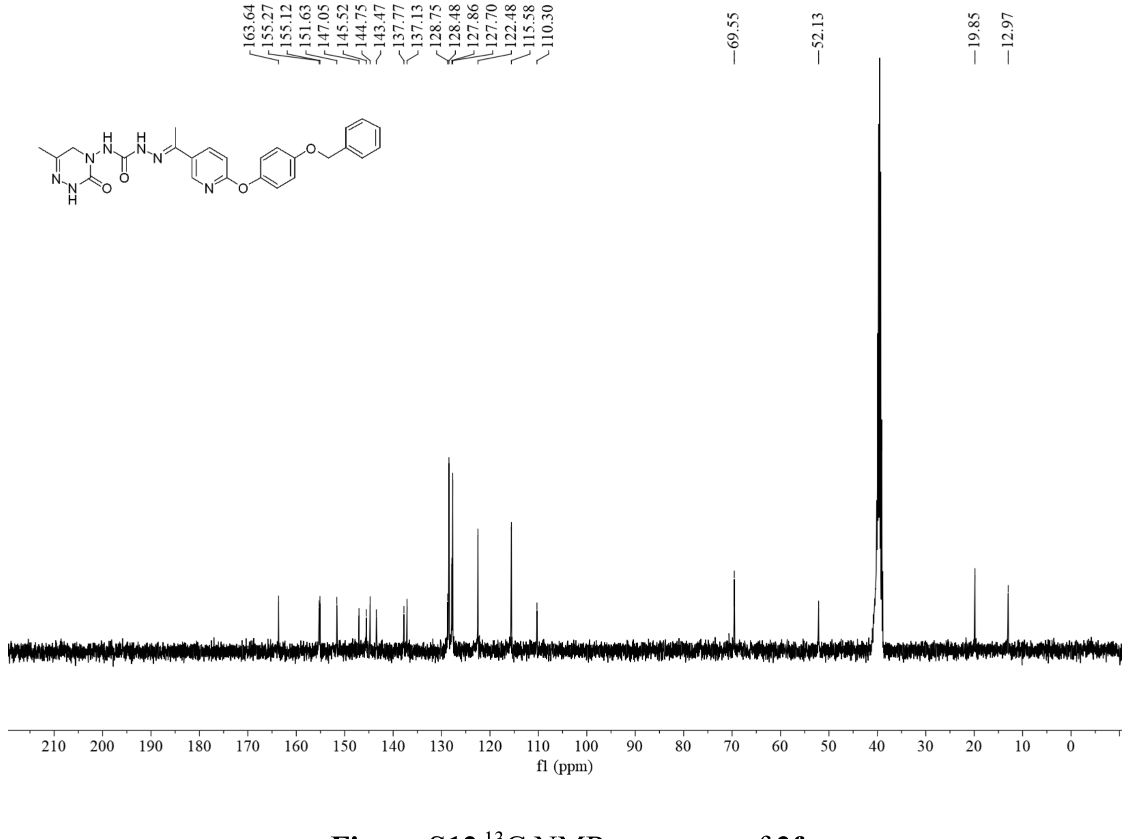

Figure S12 ^{13}C NMR spectrum of **3f**

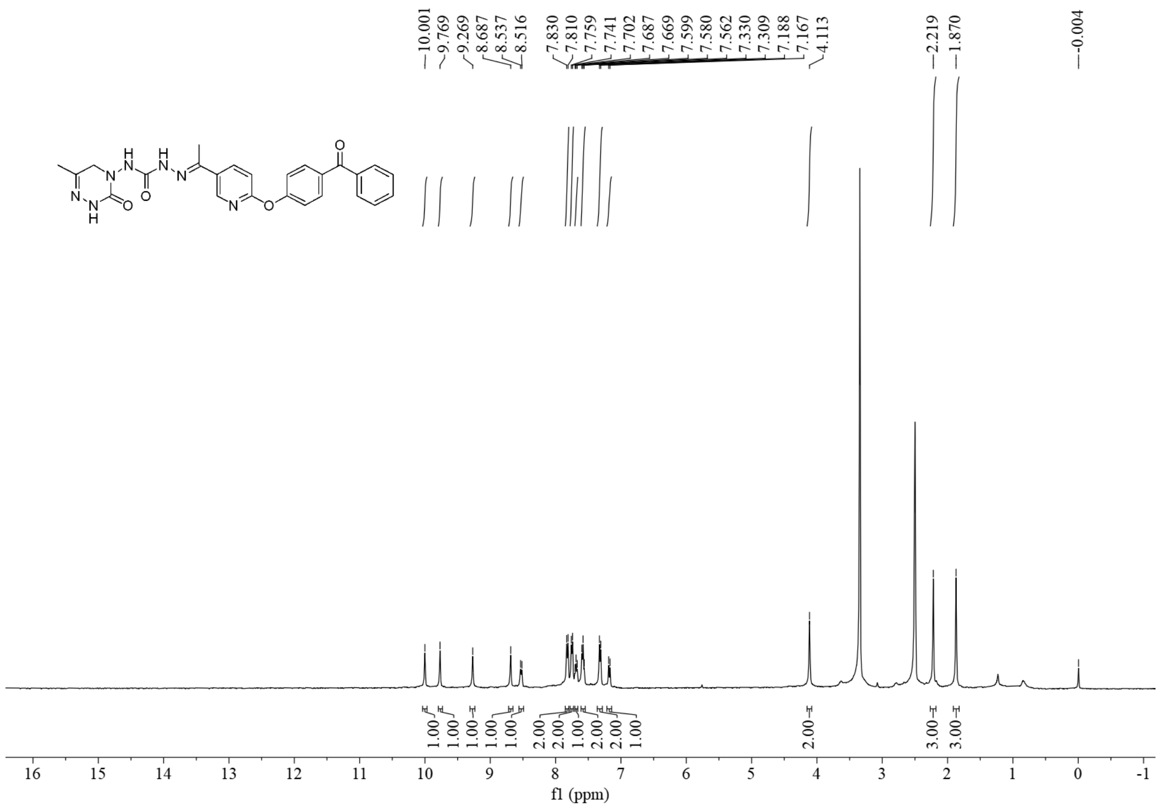

Figure S13 ^{1}H NMR spectrum of **3g**

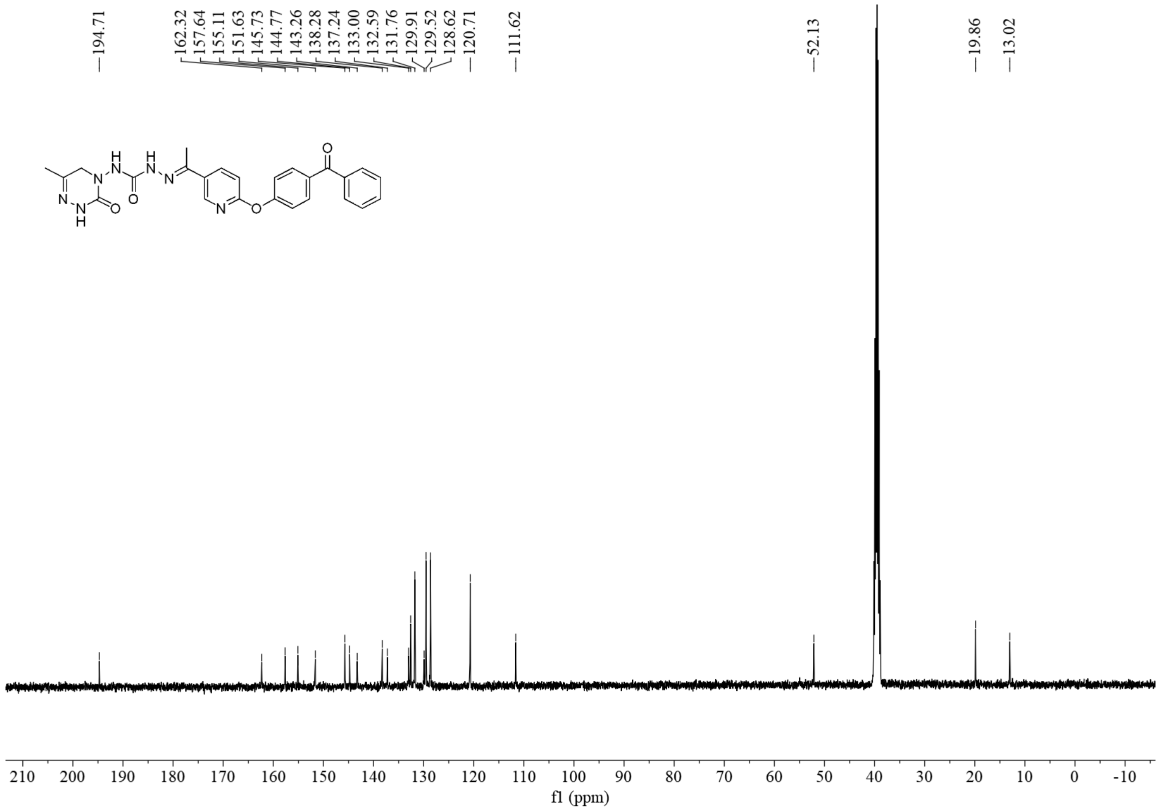

Figure S14 ^{13}C NMR spectrum of **3g**

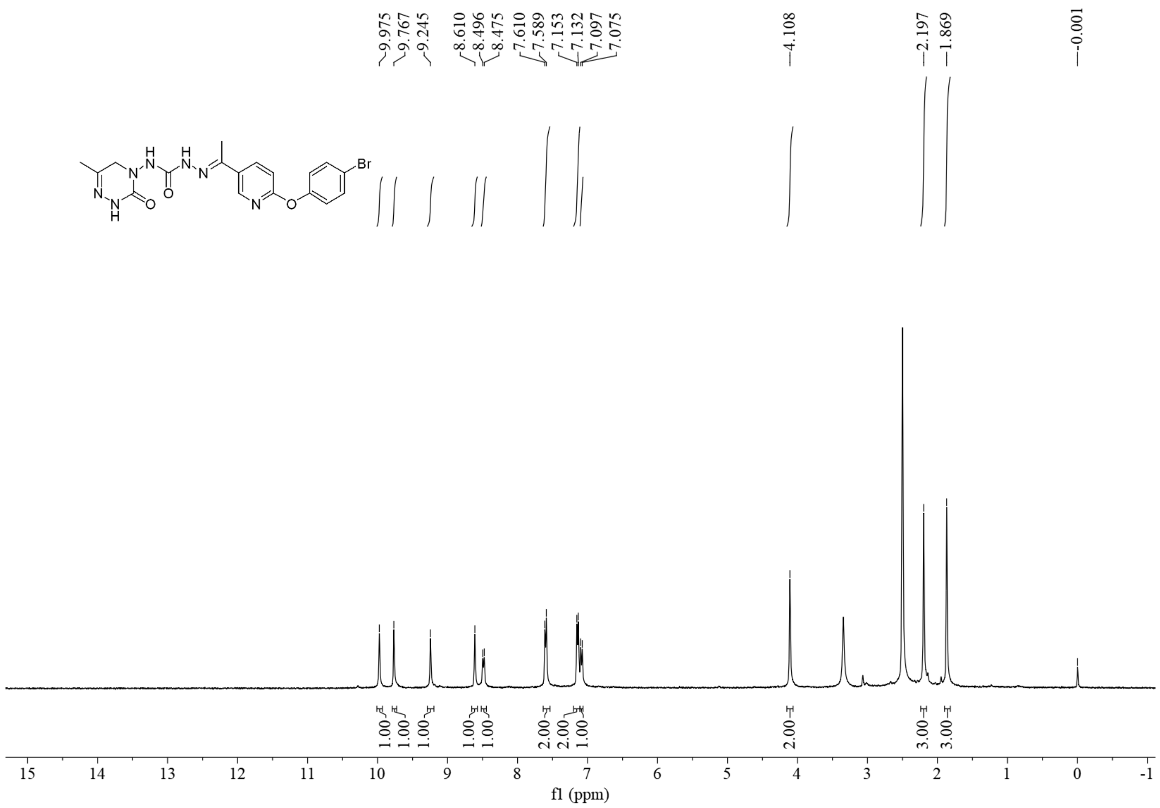

Figure S15 ^{1}H NMR spectrum of **3h**

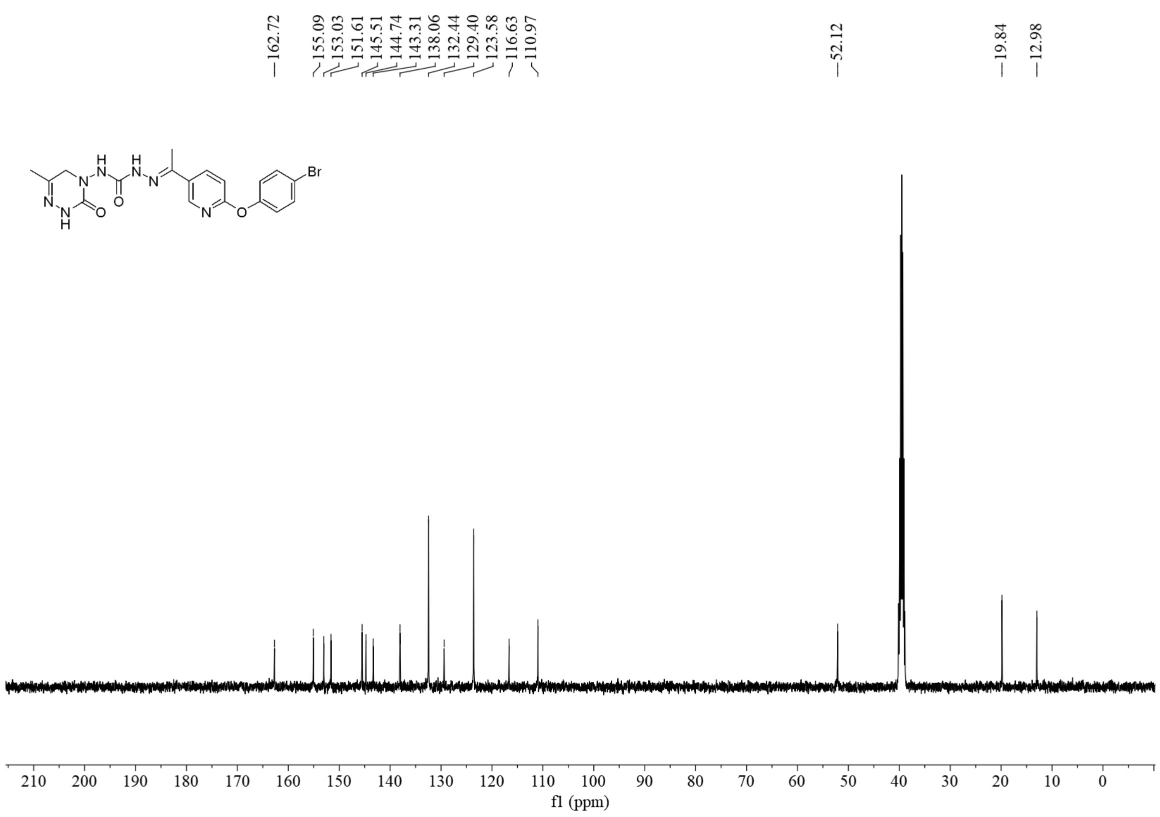

Figure S16 ^{13}C NMR spectrum of **3h**

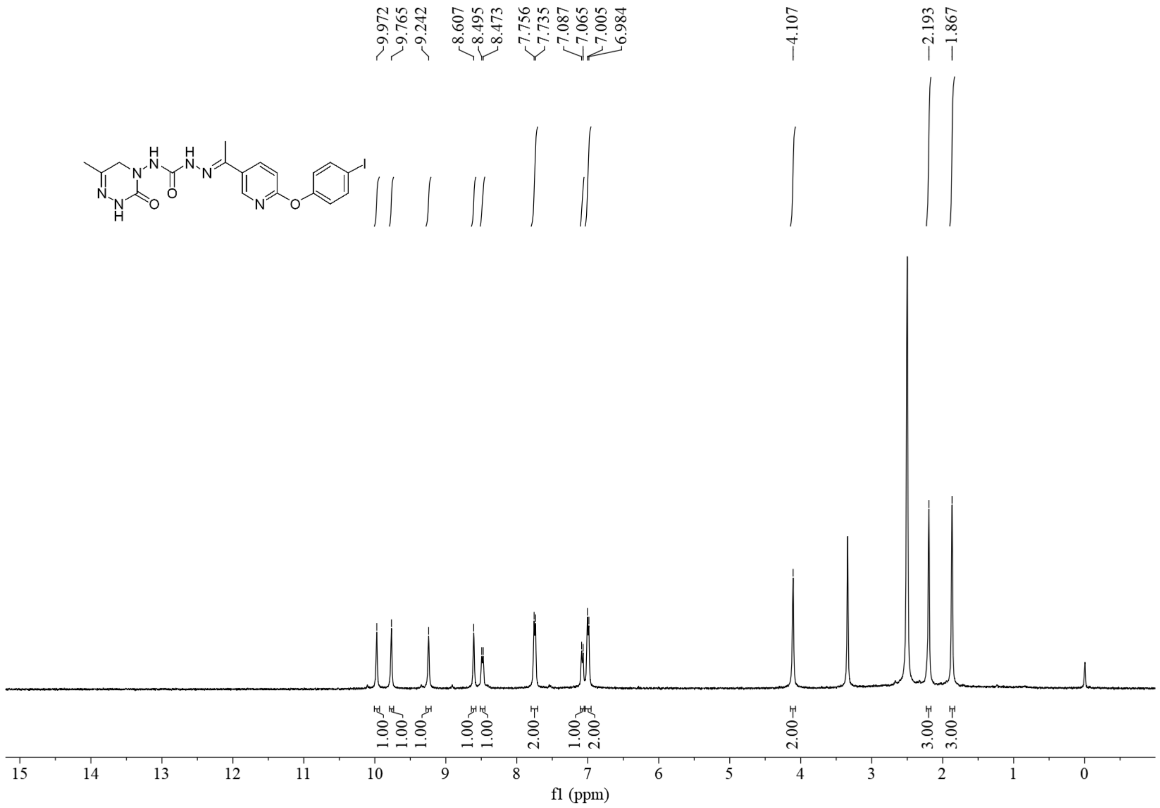

Figure S17 ^{1}H NMR spectrum of **3i**

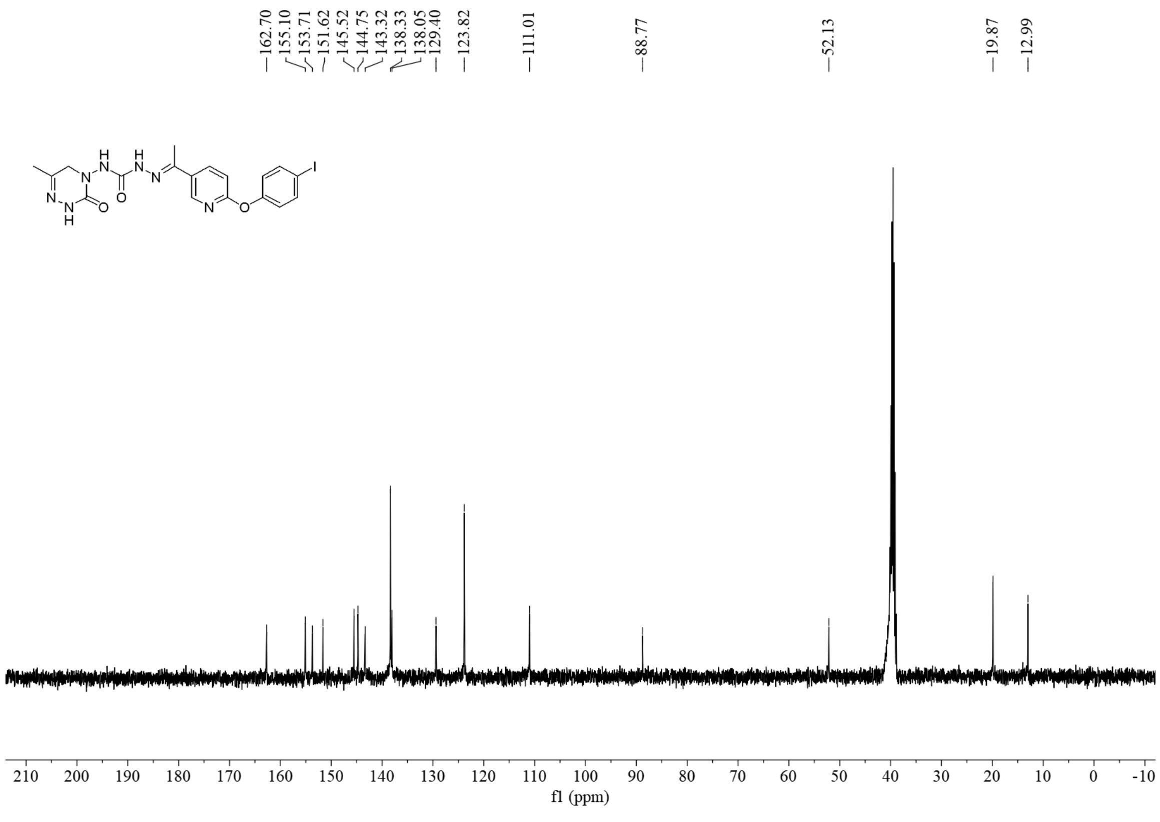

Figure S18 ^{13}C NMR spectrum of **3i**

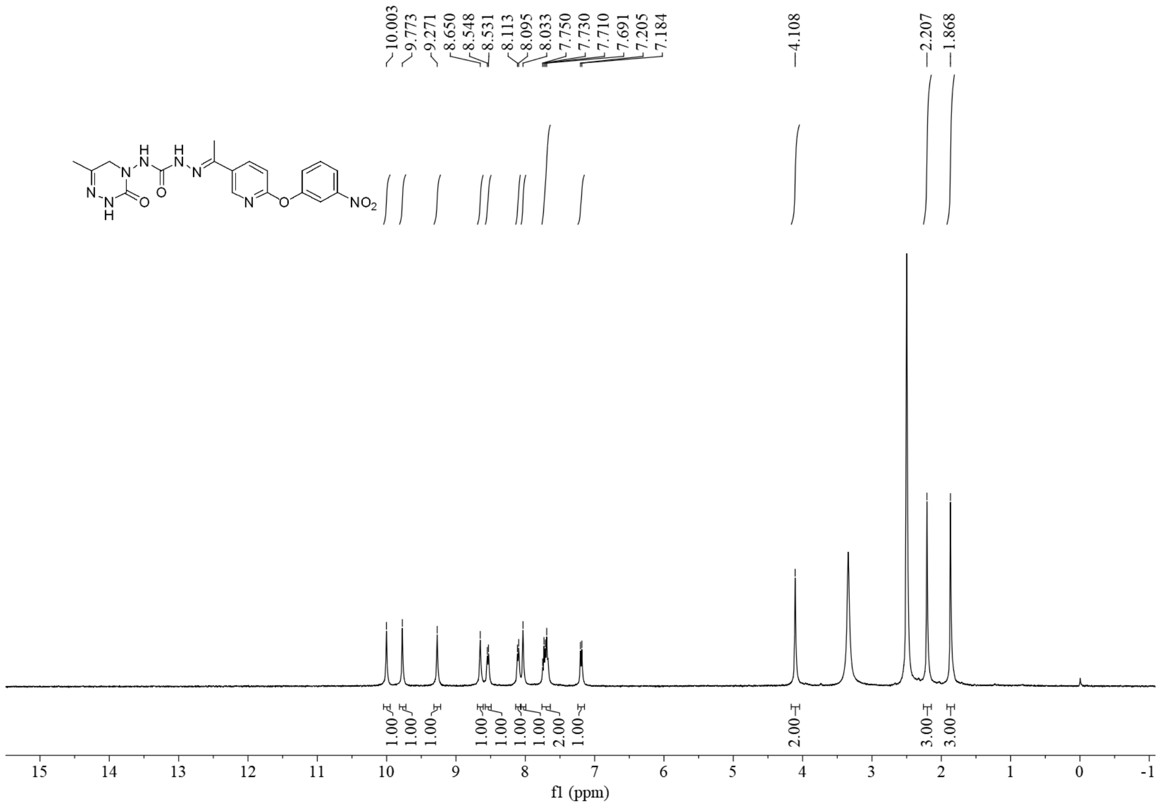

Figure S19 ^{1}H NMR spectrum of **3j**

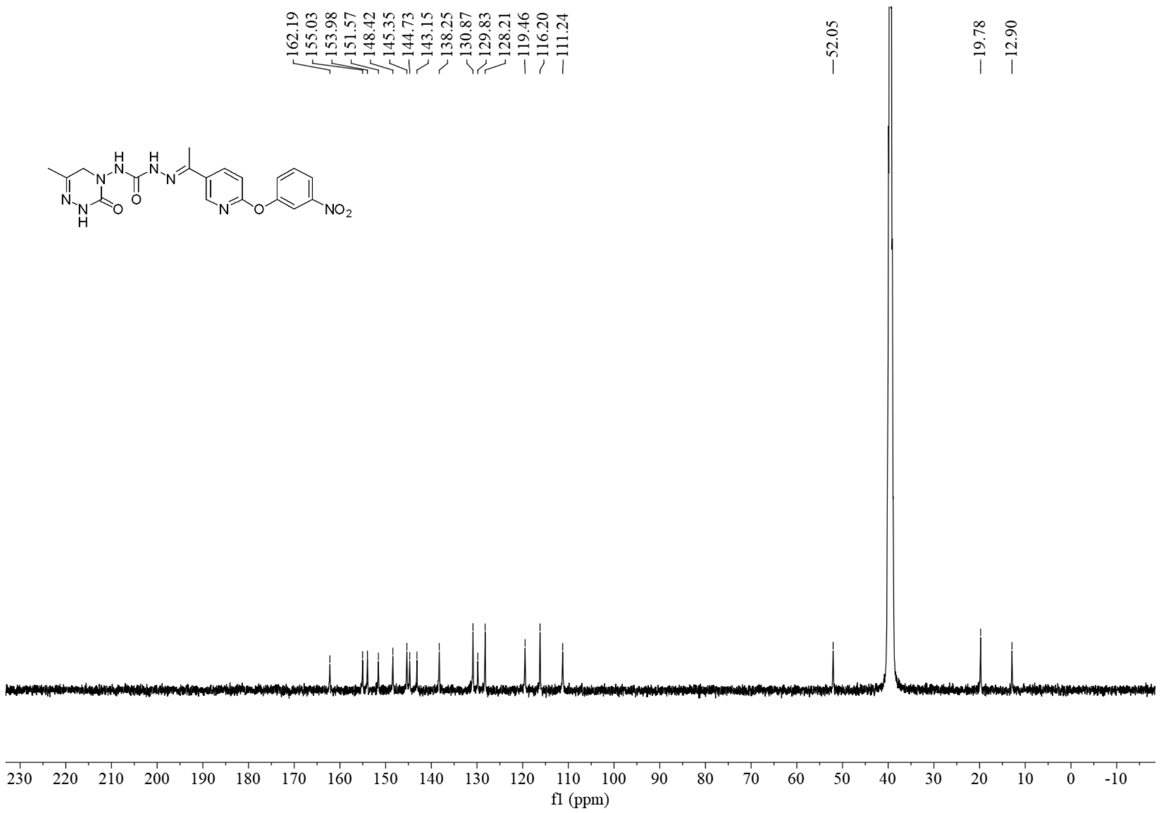

Figure S20 ^{13}C NMR spectrum of **3j**

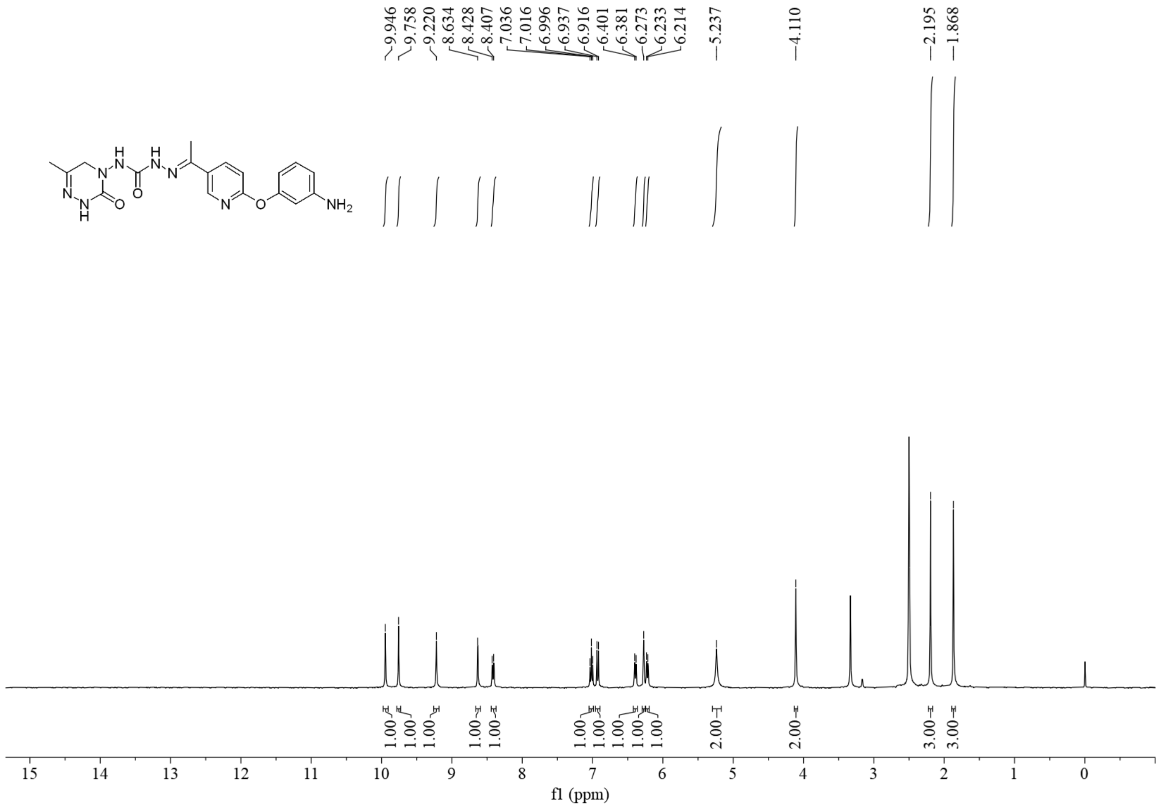

Figure S21 ^{1}H NMR spectrum of **3k**

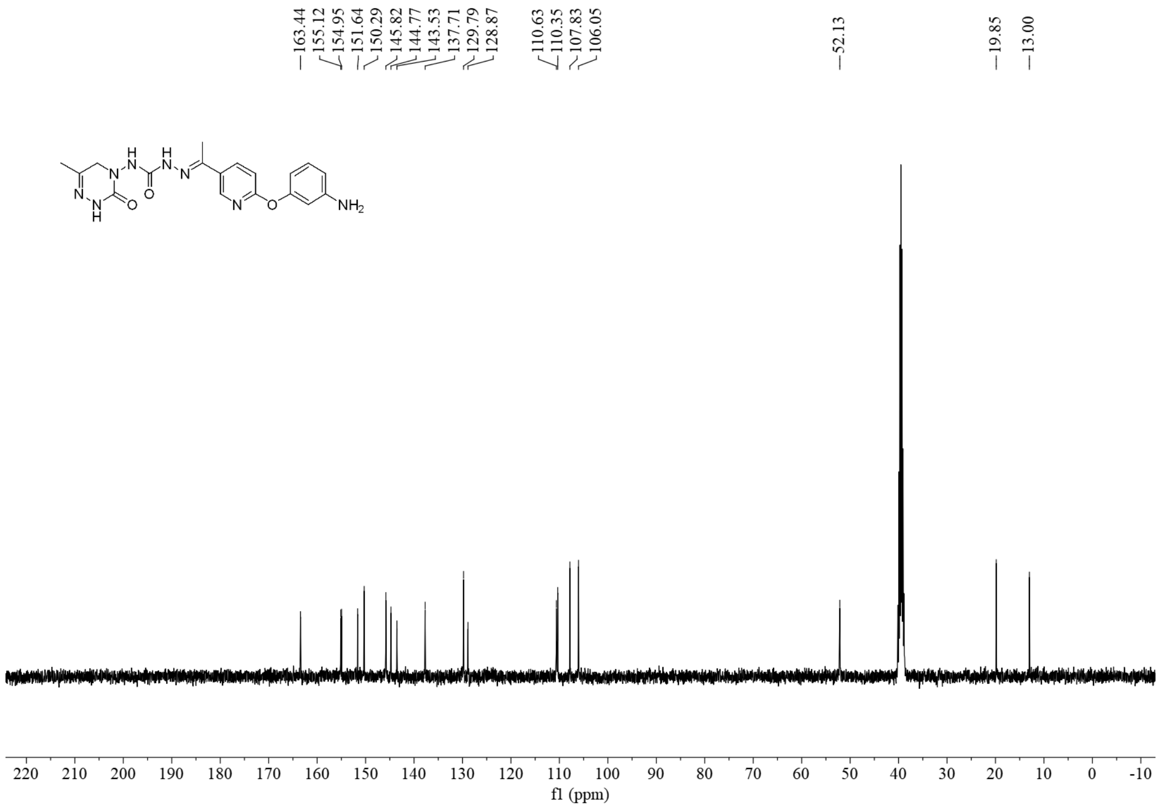

Figure S22 ^{13}C NMR spectrum of **3k**

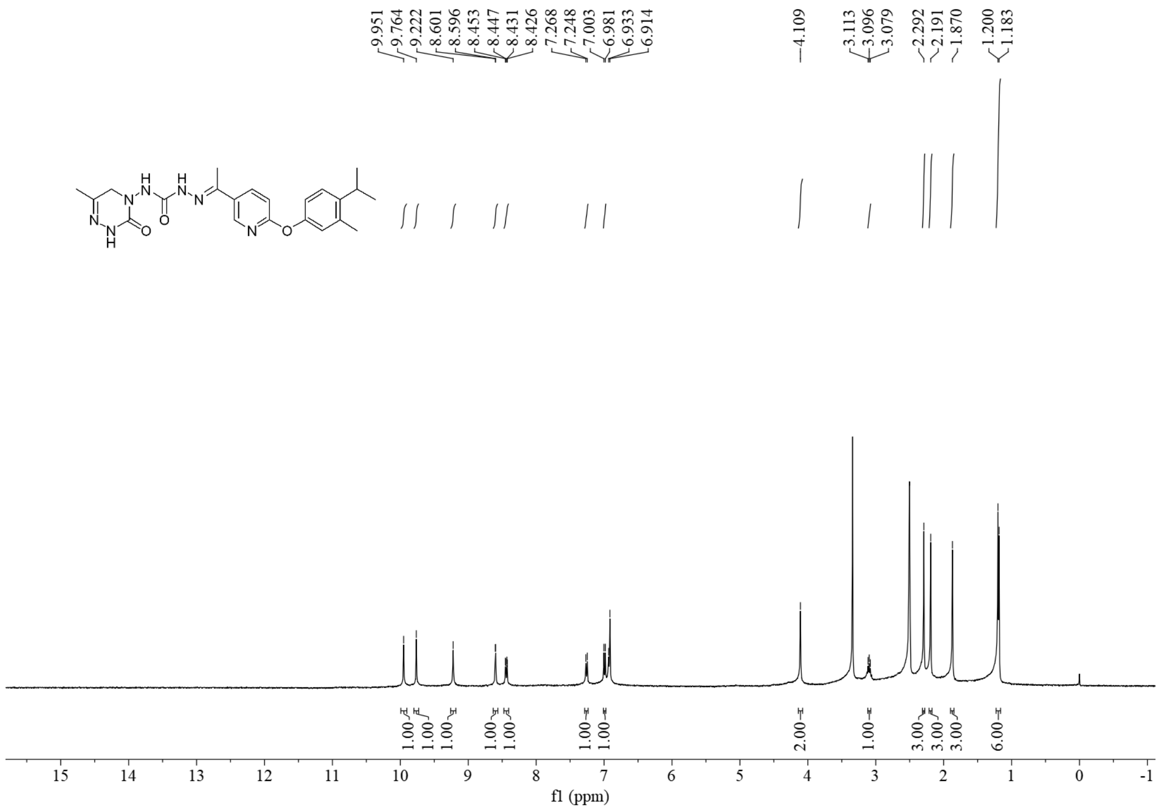

Figure S23 ^{1}H NMR spectrum of **3l**

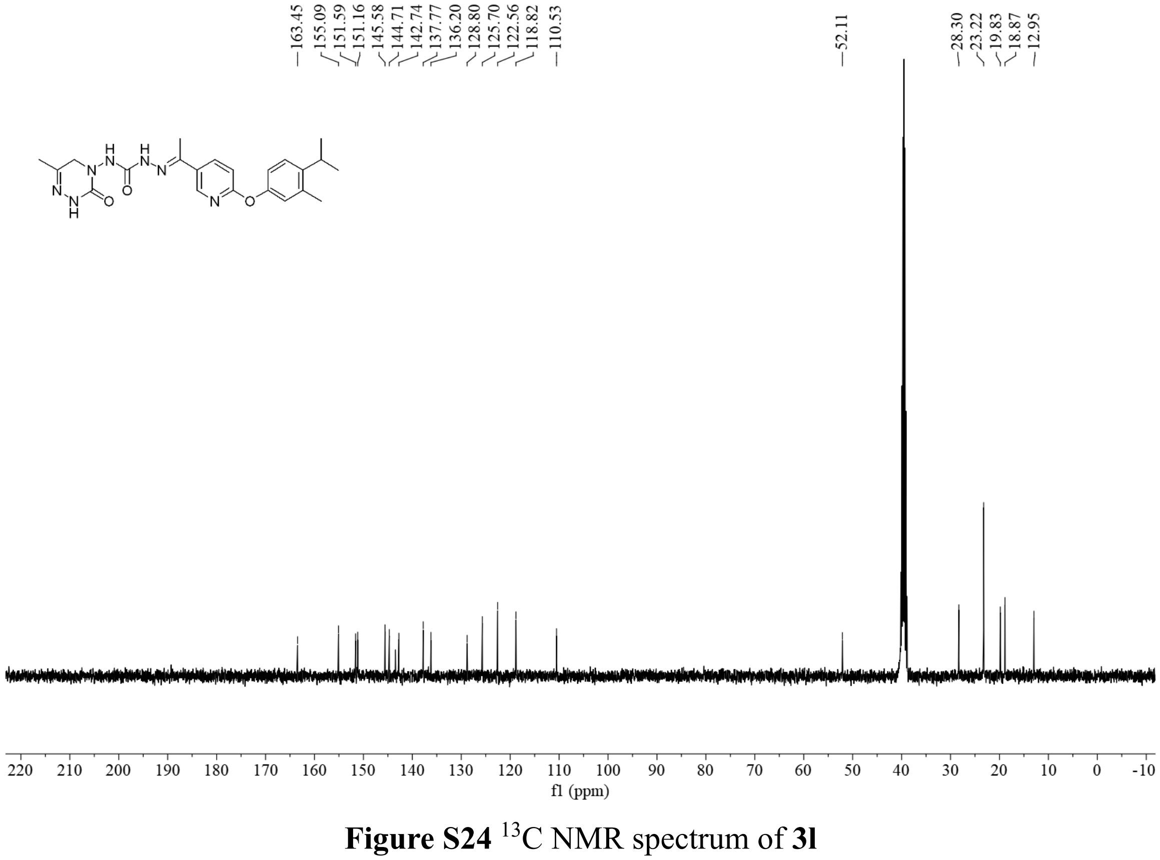

Figure S24 ^{13}C NMR spectrum of **3l**

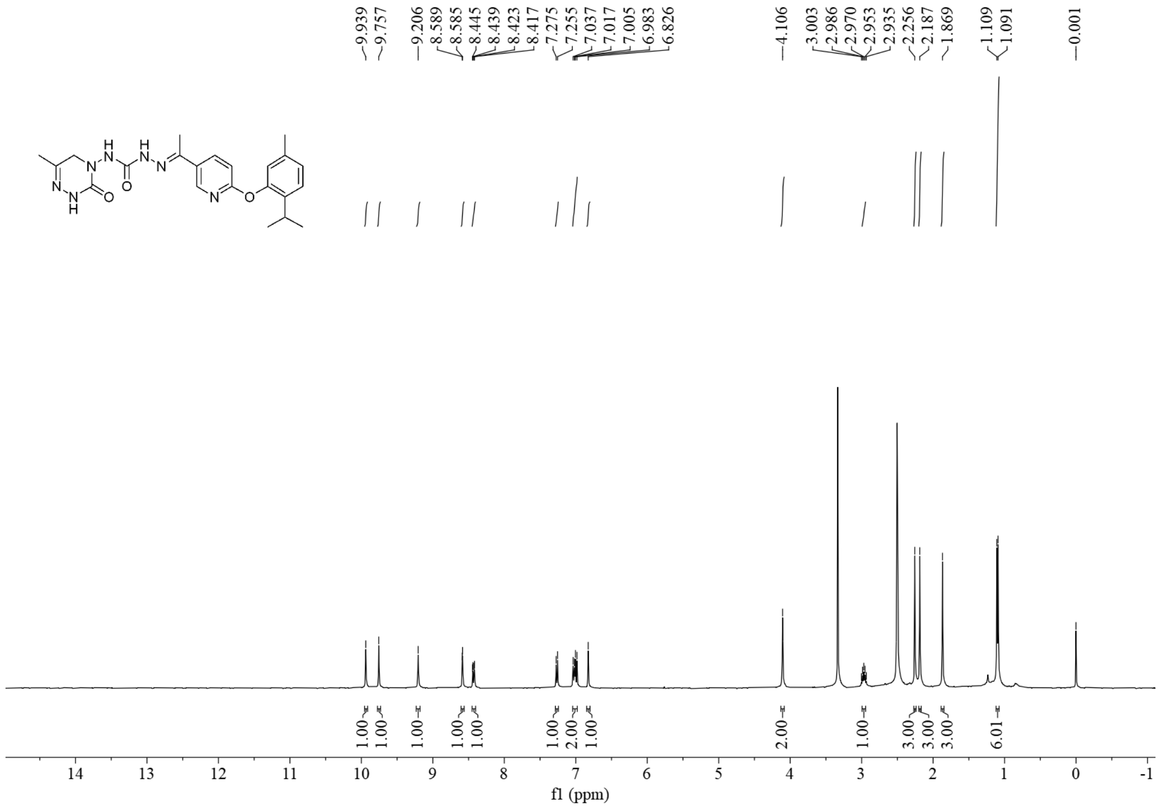

Figure S25 ^{1}H NMR spectrum of **3m**

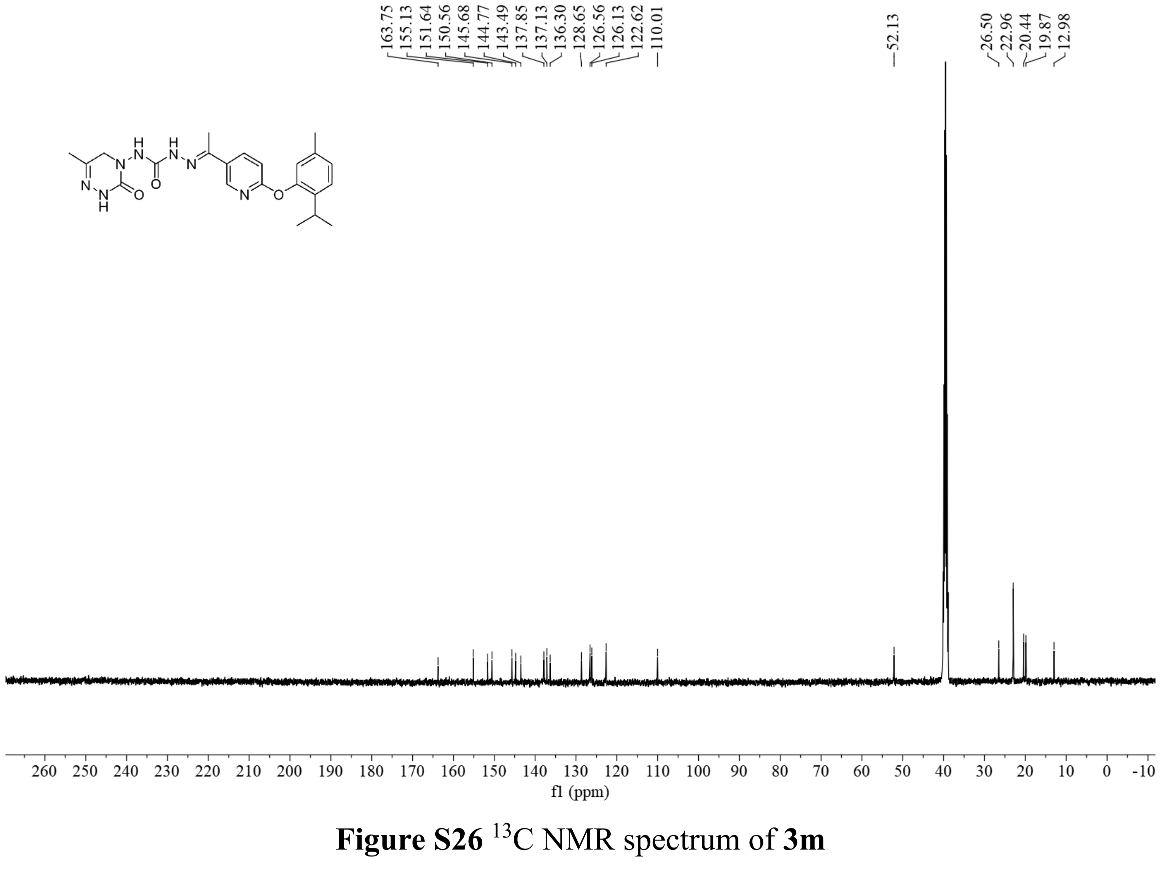

Figure S26 ^{13}C NMR spectrum of **3m**

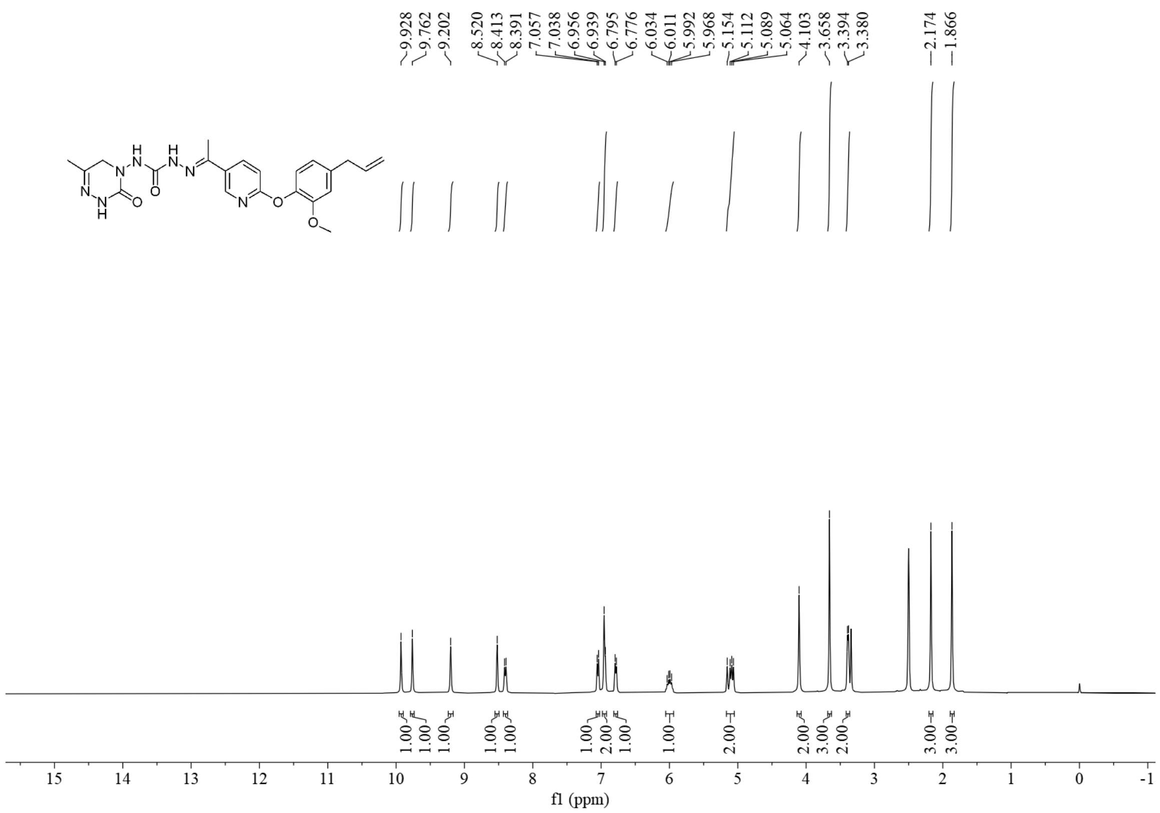

Figure S27 ^{1}H NMR spectrum of **3n**

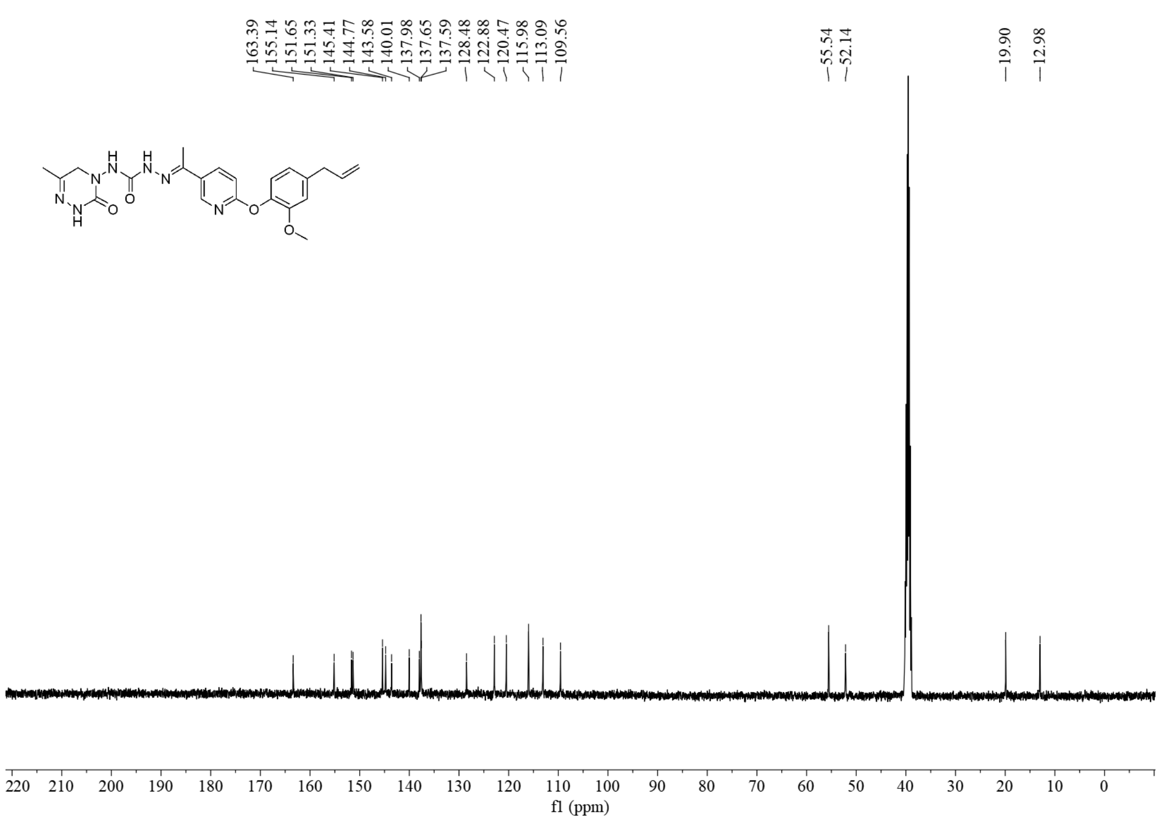

Figure S28 ^{13}C NMR spectrum of **3n**

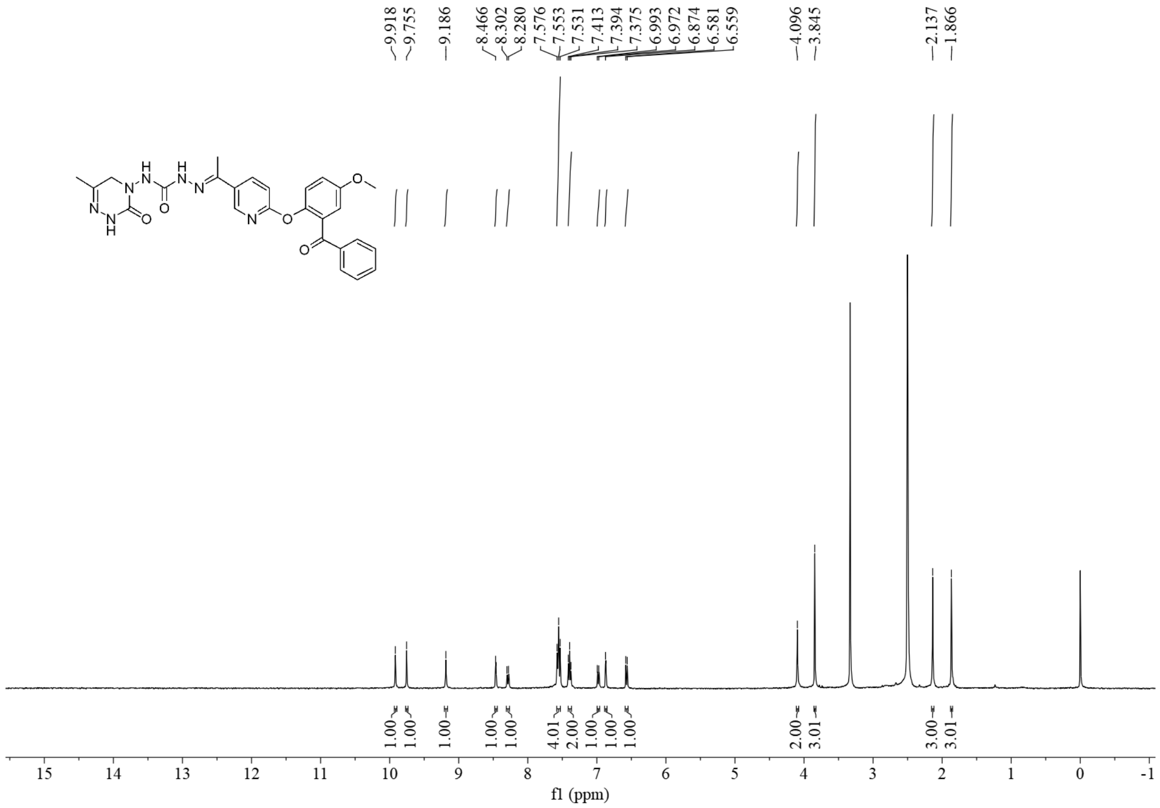

Figure S29 ^{1}H NMR spectrum of **3o**

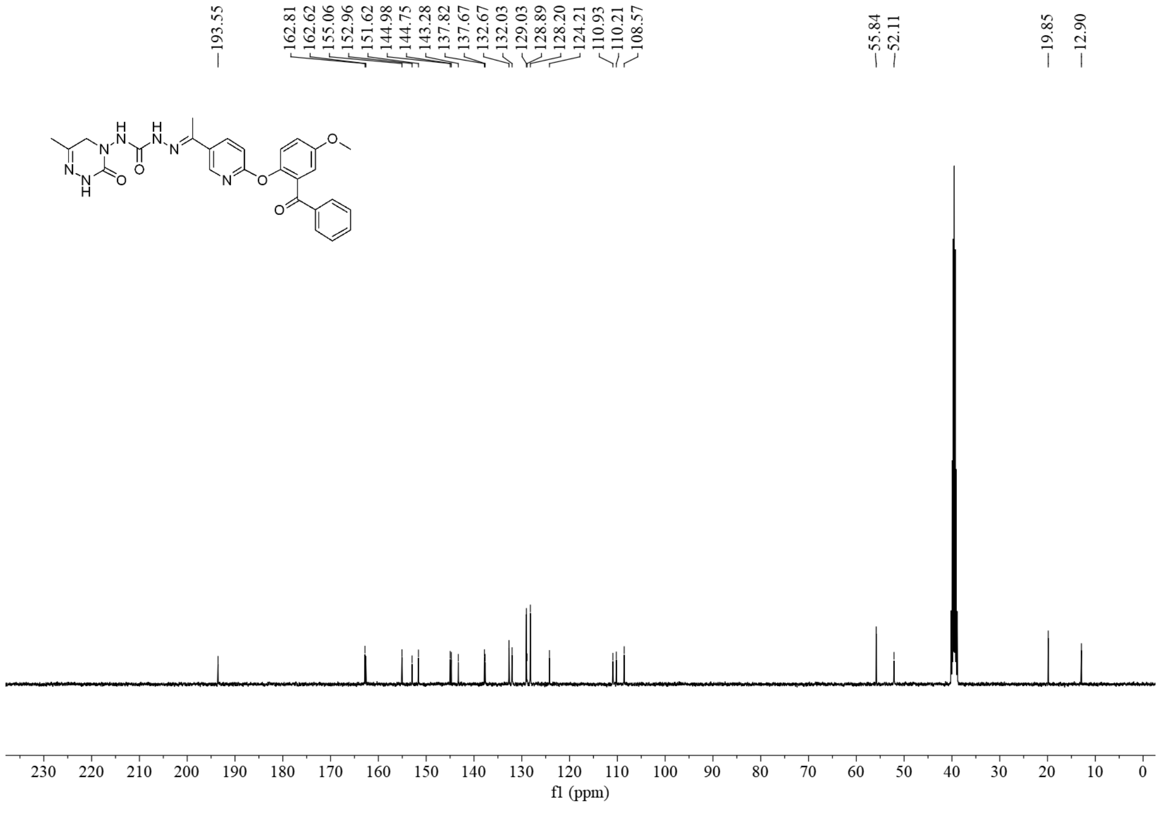

Figure S30 ^{13}C NMR spectrum of **3o**

Biological Assay

All bioassays were performed on representative test organisms reared in the laboratory. The bioassay was repeated in triplicate at 25±1 ℃. Assessments were made on a dead/alive basis, and mortality rates were corrected using Abbott's formula. Evaluations were based on a percentage scale of 0−100, where 0 equals no activity and 100 equals total kill. For comparative purpose, pymetrozine was tested under the same conditions.

Detailed bioassay procedures for the insecticidal activities.

Insecticidal activities against Aphis craccivora: The insecticidal activities of compounds 3a-3o and the pymetrozine were tested against A. craccivora by foliar application. About 60 aphids were transferred to the shoot with 3-5 fresh leaves of horsebean. The shoot with aphids was cut and dipped into the test solution for 2 s, after removing extra solutions on the leaf; the aphids were raised in the shoot at 25±1℃ and 85% relative humidity for 96 h. Each experiment for one compound was triplicated. The revised death rate was calculated by Abbott's formula.

Larvicidal Activities against cotton bollworm (*Helicoverpa armigera*), corn borer (*Ostrinia nubilalis*) and oriental armyworm (*Mythimna separata*): Stock solutions of each test compound was prepared in dimethylformamide at a concentration of 600 mg/L. Leaf-dip method was used. Leaf discs (5 cm×3 cm) were cut from fresh cabbage leaves (or other leaves) and then dipped into the test solution for 3 s. After air-drying, the treated leaf discswere placed individually into vertical tube (or Petri dishes) and the discs were infested with 10 larvae (for example: 10 second-instar diamondback moth larvae, 10

fourth-instar orientalarmyworm larvae). Percentage mortalities were evaluated 3 days after treatment. Evaluations were based on a percentage scale of 0-100, where 0 equals no activity and 100 equals total kill. Each treatment was repeated three times.

Detailed bioassay procedures for the fungicidal activities. The compounds were evaluated in mycelial growth tests in artificial media against 14 plantpathogens at rate of 50 mg/L. Test compound was dissolved in a suitable amount of acetone and diluted with water containing 0.1 % TW - 80 to the concentration of 500 mg/L. To each petri dish was added 1 mL such solution and 9 mL culture medium to make a 50 mg/L of medicated tablet, whereas to another petri dish was added 1 mL sterilized water and 9 mL culture medium as blank control. A diameter of 4 mm of hyphae was cut by a hole puncher along the hyphae for bacteria to the outerplate and moved to the medicated tablet. Each treatment was repeated three times. The dishes were stored in controlled environment cabinets (24 ± 1 ℃) for 48 h, after which the diameter of mycelia growth was investigated and percentage inhibition was calculated.

Percentage inhibition (%) = (averaged diameter of mycelia in blank controls－averageddiameter of mycelia in medicated tablets) / averaged diameter of mycelia in blank controls
